# Diet in secondary prevention: the effect of dietary patterns on cardiovascular risk factors in patients with cardiovascular disease: a systematic review and network meta-analysis

**DOI:** 10.1186/s12937-024-00922-8

**Published:** 2024-02-08

**Authors:** N. E. Bonekamp, E. Cruijsen, J. M. Geleijnse, R. M. Winkels, F. L. J. Visseren, P. B. Morris, C. Koopal

**Affiliations:** 1 Department of Vascular Medicine, University Medical Center Utrecht, PO Box 85500, Utrecht, 3508 GA the Netherlands; 2 Division of Human Nutrition and Health, Wageningen University and Research, Wageningen, Netherlands; 3 Department of Cardiology, Medical University of South Carolina, Charleston, SC USA

**Keywords:** Cardiovascular disease, Prevention, Nutrition, Dietary pattern, Preventive medicine, Network meta-analysis

## Abstract

**Background:**

Improving dietary habits is a first-line recommendation for patients with cardiovascular disease (CVD). It is unclear which dietary pattern most effectively lowers cardiovascular risk factors and what the short- and long-term effects are. Therefore, this network meta-analysis compared the effects of popular dietary patterns on cardiovascular risk factors in patients with established CVD.

**Methods:**

A systematic search of PubMed, Embase, the Cochrane library, SCOPUS and Web of Science was conducted up to 1 April 2023. Randomized controlled trials (RCTs) comparing the effect of popular dietary patterns (Mediterranean, moderate carbohydrate, low glycemic index, low-fat and minimal dietary intervention) on cardiovascular risk factors (body weight, systolic blood pressure, lipids) in CVD populations were selected. A random-effects network meta-analysis was performed.

**Results:**

Seventeen RCTs comprising 6,331 participants were included. The moderate carbohydrate diet had the most beneficial effect on body weight (-4.6 kg, 95%CrI -25.1; 15.8) and systolic blood pressure (-7.0 mmHg 95%CrI -16.8; 2.7) compared to minimal intervention. None of the included dietary patterns had a favorable effect on low-density lipoprotein cholesterol. After 12 months, the effects were attenuated compared to those at < 6 months.

**Conclusions:**

In this network meta-analysis of 17 randomized trials, potentially clinically relevant effects of dietary interventions on CV risk factors were observed, but there was considerable uncertainty due to study heterogeneity, low adherence, or actual diminished effects in the medically treated CVD population. It was not possible to select optimal dietary patterns for secondary CVD prevention. Given recent clinical trials demonstrating the potential of dietary patterns to significantly reduce cardiovascular event risk, it is likely that these effects are effectuated through alternative physiological pathways.

**Supplementary Information:**

The online version contains supplementary material available at 10.1186/s12937-024-00922-8.

## Introduction

Cardiovascular disease (CVD) remains the leading cause of mortality and morbidity worldwide, despite declining CVD mortality rates as a result of advances in diagnosis and management [1, 2]. Consequently, an increasingly large group of patients with established CVD is at risk of recurrent cardiovascular events. The impact of a healthy diet on CVD risk is hypothesized to be multifaceted, acting either as a direct and autonomous protective factor or by favorably influencing cardiovascular risk factors, such as low-density lipoprotein cholesterol (LDL-C) and systolic blood pressure (SBP) [3]. Embracing a healthy lifestyle, including healthy dietary habits, constitutes a central step in the primary and secondary prevention of CVD, an approach unanimously advocated by major CVD prevention guidelines [3, 4].

Previous meta-analyses have shown conflicting results on the effects of dietary intervention on markers of CVD, and the evidence is especially limited in patients with established CVD [5, 6]. Current treatment guidelines for CVD patients either provide dietary recommendations that are largely extrapolated from primary prevention populations or stress the need for further research [4, 7, 8]. Current guideline recommendations focus on specific food groups and nutrients rather than on dietary patterns [4, 7]. Focusing on dietary patterns may improve patients’ understanding of dietary recommendations and more adequately capture the effects of diet-related health benefits [9]. Unfortunately, the plethora of overlapping dietary patterns complicates identification of the best dietary pattern for patients with established CVD because it would require a multitude of randomized trials.

The aim of this study was to address this problem by comparing all the available randomized controlled trials on the effect of dietary patterns on cardiovascular risk factors in patients with CVD in a network meta-analysis. This approach made it possible to provide an effect estimate even when dietary pattern interventions had never been compared in a head-to-head clinical trial.

## Methods

This systematic review and network meta-analysis was prospectively registered in the PROSPERO registry for systematic reviews (CRD42021233632).

### Literature search and data extraction

A systematic literature search was performed on PubMed, Embase, the Cochrane Library, SCOPUS and Web of Science from data inception until 31 January 2022. The search was updated to cover a time range up to 1 April 2023 to ensure completeness of the results. Search terms included terms and synonyms for diet, dietary patterns, different types of CVD and RCTs. The complete search string is presented in Supplementary appendix 1.

RCTs that were performed in an adult population with established CVD (defined as a history of myocardial infarction, coronary revascularization, ischemic or hemorrhagic stroke, or peripheral arterial disease) were included. Studies were eligible if they studied the effect of an entire dietary pattern compared to an alternative dietary pattern or to a minimal dietary intervention. Changes in at least one cardiovascular risk factor over a period of at least 12 weeks should be reported as a primary or secondary endpoint. RCTs that investigated only specific food groups (*e.g.,* fruits or eggs) or specific nutrients were excluded. Studies with interventions that encompassed other components unrelated to diet, such as medication of supervised exercise, were only included if these components were applied equally to the intervention and control group (*i.e.,* both groups should receive the same medication). Eligibility was independently assessed by two authors (NEB, EC), and conflicting interpretations were resolved by consensus after inclusion of a third reviewer.

Data on study design, study population, intervention and control diet and outcomes were extracted independently by two authors (NEB, EC) using a standardized report form. Critical appraisal of the included records was independently performed by two authors (NEB, EC) using the Cochrane Risk of Bias 2 tool [10].

### Dietary pattern categories

Dietary interventions were categorized into predefined dietary pattern categories:

- Mediterranean diet: a dietary pattern rich in whole grains, green vegetables, fruits, fish, lean meat and plant-based oils.
- Low-fat diet: ≤ 30% of total energy intake from fat.
- Moderate carbohydrate diet: 30–60% of energy from carbohydrates and 10–20% of energy from protein.
- Low glycemic index (GI) diet
- Minimal dietary intervention: no changes in dietary pattern or intervention limited to pamphlet with dietary advice.

If two dietary patterns from one study were assigned to the same diet category, the findings from this study were excluded from the quantitative synthesis.

### Statistical analyses

The primary outcomes were short-term changes in body weight, SBP and LDL-C levels. Secondary outcomes were long-term changes in these three measures and short- and long-term changes in body mass index (BMI), total cholesterol, high-density lipoprotein cholesterol (HDL-C), triglyceride and C-reactive protein (CRP) levels. Short-term was defined as the measurement closest to 6 months after initiation of the dietary intervention (range 3–11 months), and long-term was defined as the first measurement at least 12 months after the start of the dietary intervention (range 12–18 months).

For each outcome, the mean change from baseline and corresponding standard deviation (SD) were extracted from the study paper. If other measures were reported (*e.g.,* standard error or means at baseline and after intervention), the mean change and SD were calculated in accordance with the Cochrane Handbook [11].

A network meta-analysis was performed to pool the available evidence. Bayesian hierarchical effect models were used to calculate a network estimate of the absolute change in cardiovascular risk factors. These estimates are presented as the effect of one diet *vs* another diet for all available comparisons. The transitivity assumption of the network meta-analysis was assessed based on study characteristics.

For each outcome, a network plot was made of the studies providing evidence, and a random-effects network meta-analysis with a Bayesian framework was calculated in a Monte Carlo Markov Chain simulation (4 chains, 5000 burn-in iterations, 100,000 iterations) [12, 13]. The convergence of the model was checked and confirmed by visual inspection of Gelman-Rubin-Brooks plots. Model fit was checked using the deviance information criterion and the posterior mean residual deviance compared to the number of data points [14]. Heterogeneity of the results was assessed using the I^2^ statistic. The consistency assumption was checked by performing node-splitting analyses to compare direct and indirect evidence and by calculating a *p* value for inconsistency.

Uncertainty surrounding the model estimates was reflected by 95% credible intervals (95% CrI) obtained from the 2.5th and 97.5th percentile values of the simulations. Ranking of the different dietary patterns was assessed in each iteration and presented as median ranking (2.5th – 97th percentile). The hierarchy of the different dietary patterns was summarized in the surface under the cumulative ranking curve (SUCRA) [15].

Sensitivity analyses were run to assess the effects of certain assumptions on the network estimates of the primary outcomes. A sensitivity analysis limited to studies published in or after the year 2000 was performed because results from older studies may not be generalizable to contemporary practice, as many pharmacotherapeutical interventions (*e.g.,* statins, blood pressure-lowering medication and platelet aggregation inhibitors) were not yet as commonly or intensively prescribed at that time. A second sensitivity analysis was performed including only studies that reported data for both the short- and long-term endpoints. The aim of this analysis was to assess whether the observed effects would be retained over a longer time span in the same population. A sensitivity analysis was limited to studies that assessed the effects of dietary interventions in CAD populations only (*N* = 15) and a final sensitivity analysis was conducted where all studies with a high risk of bias were excluded. All statistical analyses were performed using R version 4.0.4 (R Core Team, Vienna, Austria) with the gemtc package [16].

## Results

### Systematic literature search

**Fig. 1:**
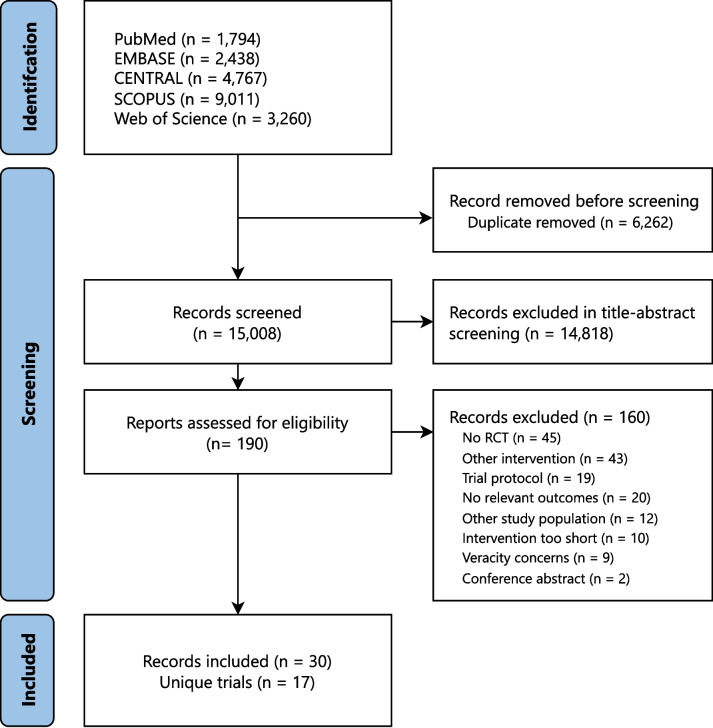
PRISMA flow diagram of study selection. This flow diagram shows the process used to identify relevant records for the network meta-analysis. The systematic literature search was performed from database inception to 1 April 2023. Abbreviations: RCT: Randomized controlled trial

The systematic literature search yielded a total of 15,008 unique publications, of which, after title and abstract screening, 190 full text records were assessed for eligibility. Ultimately, 30 records [17–46] reporting on 17 unique RCTs were included (Fig. 1). Two RCTs (Singh et al [44, 45] and von Haehling et al [46]) were excluded from the quantitative synthesis because both the intervention and control diets from these studies were classified into the same dietary category.

### Study characteristics and risk of bias assessment

Table 1 summarizes the main study characteristics of the 17 included RCTs. Additional information on the study design, population and dietary pattern interventions is presented in Table S1. Studies were published between 1965 and 2020 and comprised a total of 6,331 participants. Sixteen studies included patients with a history of coronary artery disease, and one included patients with a history of peripheral arterial disease. The median age was 61 (interquartile range (IQR) 57–62) years, and the median percentage of female participants was 17% (IQR 8–30%, range 0–61%). The use of antihypertensive and/or lipid-lowering therapy increased with more recent publications, with median percentages of 76% (IQR 53–90%) and 86% (IQR 73–87%), respectively, in studies published before and after 2000. No detailed information was available on the type of blood-pressure or lipid-lowering medications study participants used.

**Table 1:** Eligible study description and baseline study population characteristics

**Reference**	**Year**	**No**	**Intervention**	**Control**	**Follow-up** (wk)	**CVD type**	**Age** (yr)	**Female** (%)	**BMI** (kg/m^2^)	**SBP** (mmHg)	**LDL-C** (mmol/l)	**LLT** (%)	**AHT** (%)
**Ball** [17]	1965	264	Low-fat	No intervention	*159*	CAD	*NR*	*NR*	*NR*	*NR*	*NR*	*NR*	*NR*
**Oslo Diet Heart study** [18, 19]	1970	412	Moderate carb	No intervention	250	CAD	*NR*	0	*NR*	*NR*	*NR*	*NR*	*NR*
**Brown** [20, 21]	1984	50	Moderate carb	Low-fat	52	PAD	62	26	*NR*	*NR*	*4.0*	*NR*	*NR*
**Lyon Heart study** [22–24]	1994	605	Mediterranean	No intervention	117	CAD	54	9	*NR*	*120*	*4.5*	*NR*	62
**Huh** [25]	1996	14	Low-fat	No intervention	52	CAD	59	14	24.3	*NR*	*3.6*	*NR*	*NR*
**Aquilani** [26]	1999	126	Low-fat	Moderate carb	26	CAD	57	0	*NR*	*NR*	*4.6*	*NR*	*NR*
**Sondergaard** [27]	2003	131	Mediterranean	No intervention	52	CAD	63	30	26.6	*NR*	*4.0*	*NR*	*NR*
**Frost** [28]	2004	55	Low GI	No intervention	12	CAD	62	13	27.8	NR	NR	73	25
**Lindeberg** [29, 30]	2007	29	Low-fat	Mediterranean	12	CAD	61	0	29.5	NR	NR	90	NR
**This-Diet trial** [31]	2008	101	Mediterranean	Low-fat	104	CAD	58	26	*NR*	120	2.4	82	> 88
**Weber** [32]	2012	124	Moderate carb	Mediterranean	12	CAD	63	34	29.9	128	NR	87	90
**AUSMED study** [33–35]	2018	65	Mediterranean	Low-fat	52	CAD	62	16	*NR*	137	1.9	89	*NR*
**Balance** [36–38]	2019	2,521	Moderate carb	Low-fat	182	Multiple	63	42	*NR*	*NR*	*NR*	*NR*	*NR*
**DISCO-CT study** [39]	2019	81	Moderate carb	No intervention	26	CAD	60	38	29	NR	NR	69	69
**CORDIOPREV** [40–43]	2020	805	Mediterranean	Low-fat	52	CAD	60	8	31	NR	2.3	86	63
**Eligible records not included in quantitative synthesis**													
**Singh** [44, 45]	1992	406	Moderate carb	Moderate carb	36	CAD	51	10	27	*133*	*4.4*	*NR*	*NR*
**Von Haehling** [46]	2013	524	Moderate carb	Moderate carb	26	CAD	68	26	*NR*	138	3.4	58	*NR*

Characteristics of included studies. Study population characteristics refer to the entire study population (both intervention and control groups) before treatment initiation and are presented as mean (age, BMI, SBP and LDL-C) or proportion of the entire population (sex, LLT and AHT)

Additional information on study population and intervention and reference diet are provided in Table S1

*CVD* Cardiovascular disease, *BMI* Body mass index, *Moderate carb* Moderate carbohydrate, *CAD* Coronary artery disease, *PAD* Peripheral arterial disease, *SBP* Systolic blood pressure, *LDL-C* Low-density lipoprotein cholesterol, *NR* Not reported, *N* Number of included patients

The risk of bias assessment yielded a judgment of *some concerns* for the majority of the included studies, with only two records judged to be at low risk of bias (Figure S1). This risk of bias was mostly attributable to the fact that participants could not be blinded to treatment allocation and to the unavailability of prepublished study protocols available for older studies.

### Effects of dietary patterns on cardiovascular risk factors

**Fig. 2:**
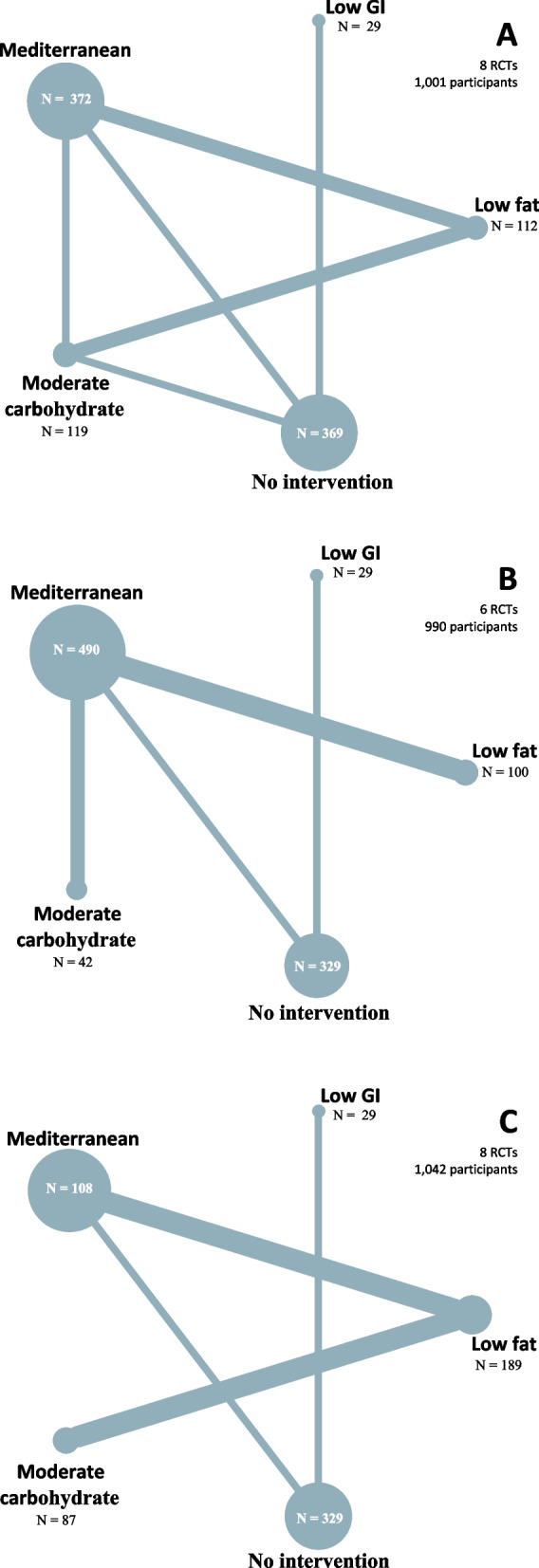
Network plots for body weight (**A**), systolic blood pressure (**B**) and LDL- cholesterol (**C**) after 6 months. For each endpoint, the number of clinical trials assessing the endpoint and number of participants are presented. The node sizes represent the number of participants randomized to a dietary pattern, and edge thickness is proportionate to the number of trials with a direct comparison between two dietary patterns. Abbreviations: RCT: randomized controlled trial, GI: glycemic index

**Fig. 3:**
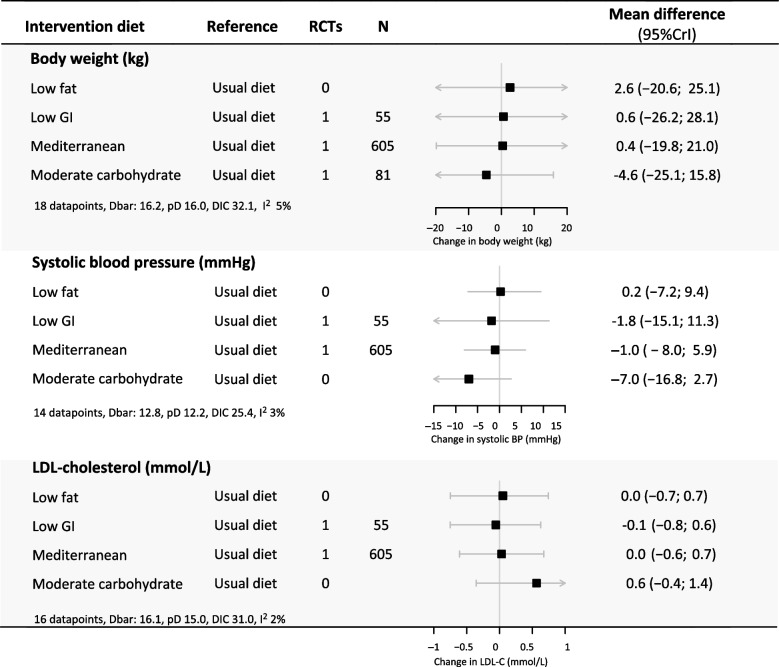
Short-term effects of dietary patterns on body weight, SBP and LDL cholesterol. This figure shows the network estimates for the 6-month difference in effect on body weight, systolic blood pressure and LDL cholesterol levels for all possible pairwise dietary comparisons. The first column shows the intervention diet and the second column shows the reference diet. The column ‘Direct comparisons’ presents the number of trials in which a diet was directly compared to another diet, and the ‘N’ column presents the total number of participants included in these trials. Zero direct comparisons mean that the presented network estimate is based on indirect evidence alone. Abbreviations: RCT: randomized controlled trial, CI: credible interval, Moderate carb: moderate carbohydrate, SBP: systolic blood pressure, LDL-C: low-density lipoprotein cholesterol

Figures 2, S2 and S3 show the networks of eligible studies for the primary and secondary outcomes. Compared to the minimal change diet, the moderate carbohydrate diet showed the largest reductions in body weight (-4.6 kg, 95%CrI-25.1;15.8) and SBP (-7.0 mmHg, 95%CI -16.8; 2.7), while increasing LDL-C (0.6 mmol/L, 95%CrI -0.4; 1.4). None of the dietary patterns lowered LDL-C compared to a minimal dietary intervention. The results for all pairwise comparisons can be found in Figure S5. The moderate carbohydrate pattern had the best ranking for body weight and SBP, and the low GI diet ranked best for LDL-C (Figure S6). None of the dietary patterns had a statistically significant short-term effect on body weight, BMI, SBP or LDL-C (Figs. 3 and S4).

**Fig. 4:**
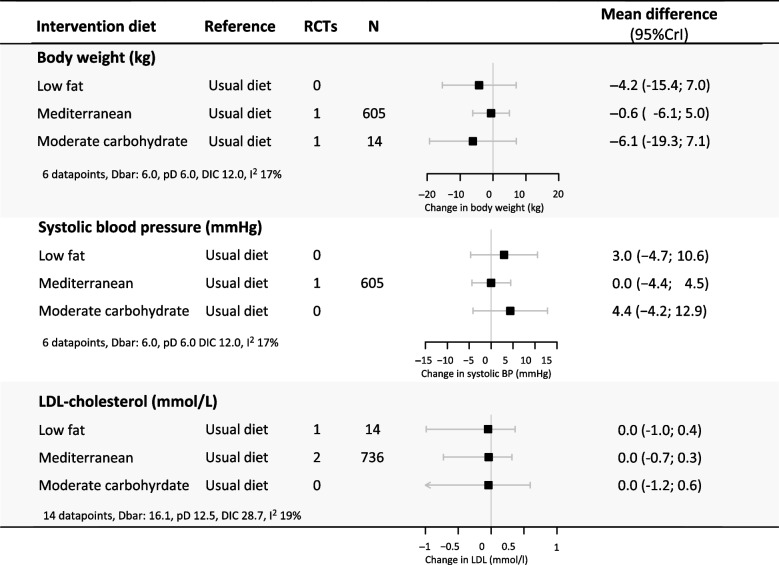
Long-term effects of dietary patterns on body weight, SBP and LDL cholesterol. This figure shows the network estimates for the 12-month difference in effect on body weight, systolic blood pressure and LDL cholesterol levels for all possible pairwise dietary comparisons. The first column shows the intervention diet and the second column shows the reference diet. The column ‘Direct comparisons’ presents the number of trials in which a diet was directly compared to another diet, and the ‘N’ column presents the total number of participants included in these clinical trials. Zero direct comparisons mean that the presented network estimate is based on indirect evidence alone. Abbreviations: RCT: randomized controlled trial, CI: credible interval, Moderate carb: moderate carbohydrate, SBP: systolic blood pressure, LDL-C: low-density lipoprotein cholesterol

At > 12 months, the moderate carbohydrate and low-fat diets resulted in a decrease in body weight (-6.1 kg (95%CrI-19.3;7.1) and -4.2 kg (95%CrI 15.4;7.0), respectively) and an increase in SBP (4.4 mmHg (95%CrI -4.2;12.9) and 3.0 mmHg (95%CrI -4.7;10.6), respectively) compared to minimal dietary intervention. The results for all pairwise comparisons and corresponding rankings are presented in Figures S5 and S6. No statistically significant long-term (> 12 months) differences were observed with all head-to-head comparisons (Fig. 4). Figure S5 shows the corresponding ranking of the different treatments.

For the secondary outcomes, the low-fat diet had the most beneficial effect on total cholesterol (-0.6 mmol/l, 95%CrI -1.4; 0.3, Figure S5). The low GI diet decreased triglycerides the most (-0.3 mmol/l, 95%CrI -1.2; 0.6). A low-fat diet reduced CRP compared to a Mediterranean diet (-0.3 mg/L, 95%CrI -1.4;0.6), but only one study was avaialble. None of the effects on secondary outcomes was statistically significant for the short or long term (Figure S4).

### Sensitivity analyses

After excluding studies published before 2000, the effects on body weight, SBP and LDL-C were comparable to those found in the complete dataset (Figure S6). Only three RCTs published after 2000 reported an effect on LDL-C levels, and the results were similar to those of the RCTs published before 2000. The tendency toward an increasing effect of a moderate carbohydrate diet on LDL-C was not seen in this sensitivity analysis.

In networks comprising only studies that reported on both short- and long-term effects, overall, the results were similar. However, the low-fat diet showed a tendency to decrease body weight and increase SBP, both in the short and long term. In particular, the short-term effect of a low-fat diet on body weight was larger than that in the main analysis (Figure S7). In this subset, LDL-C levels were similar in both the long and short term, although they were closer to zero in the long term (Figure S7). Sensitivity analysis limited to patients with CAD or excluding studies with a high risk of bias yielded non-statistically significant results, similar to the main analysis (Figures S8 and S9).

## Discussion

In this systematic review and network meta-analysis of 17 RCTs comprising 6,331 patients with established CVD, no significant effect on cardiovascular risk factors was found for interventions with a low-fat, Mediterranean, low GI or moderate carbohydrate dietary pattern compared to a minimal dietary intervention in patients with CVD.

This study is the first network meta-analysis on dietary patterns in patients with CVD. However, similar analyses have been performed in populations at high risk of CVD. These studies showed similar effect estimates for different CVD risk factors, albeit with smaller confidence intervals and more statistically significant findings [47, 48]. One network meta-analysis in overweight and obese populations showed that low carbohydrate, low-fat and moderate macronutrient diets were associated with clinically relevant and statistically significant reductions in body weight up to -4.6 kg and blood pressure up to -5.0 mmHg compared to the usual diet [47]. Our study showed similar short-term effects for the moderate carbohydrate pattern on SBP, but not body weight, and smaller effects for other dietary patterns. Another network meta-analysis in patients with type 2 diabetes showed that the Mediterranean diet was effective in reducing body weight and LDL cholesterol, but no effect of other dietary patterns was found [49].

There are multiple potential explanations for the wide credibility intervals around the effects of dietary patterns on CVD risk factors. First, it is conceivable that the effect of diet is smaller in CVD populations than in patients without CVD because the administration of lipid-lowering and antihypertensive medications to CVD patients might limit the potential influence of dietary interventions on lipid and blood pressure profiles.

Second, the quantity and quality of the available evidence might be insufficient to find a statistically significant effect. Fifteen of 17 included studies were judged as ‘some concerns’ in the risk of bias assessment, specifically stemming from the inability to blind participants to the intervention. This may have inadvertently led patients in the control groups to adopt certain components of the intervention in the intervention group, which leads to an underestimation of the effect. Additionally, the number of trials available for this network meta-analysis may have been too small to show statistically significant evidence. For example, although we found similar effects of a moderate carbohydrate dietary pattern on SBP as studies in persons without CVD [47], our results had wide credibility intervals and were therefore not statistically significant.

A third explanation for the absence of an effect might be rooted in low adherence to the dietary patterns in the included trials. Although the majority of the included RCTs did not explicitly measure or report adherence to the dietary pattern intervention, it is reasonable to assume that adherence decreased over time. Low compliance rates are a major issue, especially in long-term dietary trials, where a significant proportion of participants return to their original dietary pattern [48] if not regularly counseled [50]. Therefore, strategies to increase adherence may be more important for obtaining meaningful cardiovascular benefits than the specific macronutrient composition of the dietary pattern itself.

Fourth, amelioration of traditional risk factors probably does not capture the full effect of dietary interventions on cardiovascular event risk. Alternative pathways could be low-grade inflammation, lipid composition and vascular function. Moreover, the cumulative effect of small improvements in individual risk factors may translate into a more considerable decrease in overall CVD risk. Further research is needed to elucidate the mediating pathways between dietary pattern interventions and cardiovascular risk reduction.

The findings of the current study stand in contrast with previous observational and experimental studies on the relationship between dietary patterns and occurrence of cardiovascular events, especially on the Mediterranean diet [51]. Long-term randomized controlled trials such as the Lyon diet heart study, the PREDIMED study and the CORDIOPREV trial demonstrated a beneficial effect of a Mediterranean diet compared to low-fat diets in both primary and secondary CVD prevention populations and resulted in 30–50% relative risk reduction of (recurrent) cardiovascular events [24, 43, 52]. Interestingly the CORDIOPREV trial found non-significant changes in intermediate endpoints such as weight, LDL-cholesterol en systolic blood pressure [43]. The present study also suggests that the beneficial effects of dietary patterns may not primarily be achieved through reduction of such traditional cardiovascular risk factor levels. While the diets included in this analysis have different macronutrient distributions, generally they all recommend sufficient fruit and vegetable intake and consumption of fish and other products rich in unsaturated fatty acids. These dietary components are known to mitigate systemic inflammation, offering a plausible explanation for the discordance between the current findings and those of previous long-term studies [53]. Moreover, the emphasis on home-cooked, whole-food items within nearly all dietary patterns in this network meta-analysis could elucidate why none outperformed the others. In contrast, diets rich in ultra-processed foods, characterized by low nutritional value and elevated sodium and trans-fatty acid content, have been associated with an increased risk of cardiovascular disease [54, 55].

For the implementation of dietary interventions in the clinical management of CVD patients, a shift towards assessing their impact on cardiovascular event risk is recommended, as opposed to focusing solely on cardiovascular risk factor levels. The current analysis underscores that efficacy with regard to these endpoints does not directly align with efficacy on cardiovascular event risk. Furthermore, in patients with established CVD, the inclusion of non-traditional cardiovascular risk factors such as CRP as trial outcomes in dietary pattern trials should be considered as these could provide more informative insights into efficacy concerning cardiovascular event risk [43].

Strengths of this study include the systematic literature search and the selection of only RCTs to limit the impact of bias. Moreover, network meta-analysis techniques allowed for combining direct and indirect evidence, which increases power and enables comparison of interventions that have not been directly compared in an RCT. Finally, a wide range of cardiovascular risk factors was examined. Study limitations include that some outcome measures had to be manually calculated because not all included studies reported their outcomes as the mean differences, and these calculations may be less accurate. We used strict selection criteria in the systematic literature search to ensure applicability of the results to our target population, but as a result, few studies met the eligibility criteria, leading to wide credibility intervals. Furthermore, the limited number of studies prevented extensive sensitivity and subgroup analysis, such as analyses to assess the impact of sex, concomitant use of medication or the type of counselling provided in the included trials. Ethnic background may affect the metabolic response to dietary intervention [56], but the majority of studies included in this analysis (13 out of 17) were conducted in European and North American countries and ethnicity was rarely reported. This may limit the generalizability of the findings to populations in other countries. Finally, the included studies showed considerable heterogeneity in study characteristics, such as sex distribution and medication use, and the limited number of RCTs prevented adjustment for such sources of heterogeneity. However, careful node-splitting analyses gave no indication that the consistency assumption was violated, meaning that the heterogeneity between different RCTs did not result in conflicts between direct and indirect evidence.

To solidify the role of dietary counseling in clinical care for patients with established CVD, further research is necessary to adequately assess the effect of dietary patterns and their mediating pathways in preventing CVD outcomes in patients with CVD. Another point of interest should be how to improve long-term adherence to a healthy dietary pattern.

## Conclusion

In this network meta-analysis of 17 randomized trials, there was not one single best dietary pattern, and long-term effects were attenuated. The nonsignificant findings might be explained by inadequate sample size, diminished dietary effects in CVD populations, low adherence, or mediation by other pathways. To answer this question, more randomized trials are needed that focus on the protective effects of diet on CVD outcomes in CVD populations.

### Supplementary Information

**Additional file 1: Supplementary appendix 1.** Search strategy. **Table S1.** Baseline characteristics of studies included in the systematic review and network meta-analysis. **Figure S1.** Risk of Bias assessment. **Figure S2.** Network plots for short-term outcomes. **Figure S3.** Network plots for long-term outcomes. **Figure S4.** League tables of the network estimates for the short- and long- term effects of dietary pattern on cardiovascular risk factors. **Figure S5.** Short term effects SUCRA values for all outcomes. **Figure S6.** Sensitivity analysis: 6-month effects on body weight, systolic blood pressure and LDL-C in studies published in or after 2000. **Figure S7.** Sensitivity analysis: Comparison of short- and long term effects on primary outcomes. **Figure S8.** Sensitivity analysis - League tables for 6-month change in body weight, systolic blood pressure and LDL-cholesterol limited to CAD patients. **Figure S9.** Sensitivity analysis - League tables for 6-month change in body weight, systolic blood pressure and LDL-cholesterol after exclusion of studies judged to be at high risk of bias.

## Data Availability

Data described in the manuscript, code book, and analytic code will be made available upon request pending application and approval from the corresponding author.
