# Supplementary material for: Diet in secondary prevention: the effect of dietary patterns on cardiovascular risk factors in patients with cardiovascular disease: a systematic review and network meta-analysis
Source: Nutr J. 2024 Feb 8;23:18. doi: 10.1186/s12937-024-00922-8 (PMC10851459; doi:10.1186/s12937-024-00922-8)
Supplement: Supplementary file 1 — Additional file 1: Supplementary appendix 1. Search strategy. Table S1. Baseline characteristics of studies included in the systematic review and network meta-analysis. Figure S1. Risk of Bias assessment. Figure S2. Network plots for short-term outcomes. Figure S3. Network plots for long-term outcomes. Figure S4. League tables of the network estimates for the short- and long- term effects of dietary pattern on cardiovascular risk factors. Figure S5. Short term effects SUCRA values for all outcomes. Figure S6. Sensitivity analysis: 6-month effects on body weight, systolic blood pressure and LDL-C in studies published in or after 2000. Figure S7. Sensitivity analysis: Comparison of short- and long term effects on primary outcomes. Figure S8. Sensitivity analysis - League tables for 6-month change in body weight, systolic blood pressure and LDL-cholesterol limited to CAD patients. Figure S9. Sensitivity analysis - League tables for 6-month change in body weight, systolic blood pressure and LDL-cholesterol after exclusion of studies judged to be at high risk of bias. [file 12937_2024_922_MOESM1_ESM.pdf]

Supplemental material to

Diet in secondary prevention: the effect of dietary patterns on cardiovascular risk factors in patients with cardiovascular disease.

A systematic review and network meta-analysis

N.E. Bonekamp, E. Cruijsen, J.M. Geleijnse, R.M. Winkels,

F.L.J. Visseren, P.B. Morris, C. Koopal

Supplementary appendix 1 Search strategy

The presented search string was used in PubMED from database inception until April 1, 2023. The same search string was used in EMBASE, The Cochrane library, Web of Science and SCOPUS after alterations were made to meet their respective search engines.

("alkaline"[Title/Abstract] OR "atkins"[Title/Abstract] OR "biggest loser"[Title/Abstract] OR "bulletproof"[Title/Abstract] OR ("DART"[Title/Abstract] OR "Reinfarction trial"[Title/Abstract]) OR ("DASH"[Title/Abstract] OR "Dietary approach to stop hypertension"[Title/Abstract]) OR "drinking man*"[Title/Abstract] OR "Dukan"[Title/Abstract] OR "Engine"[Title/Abstract] OR ("F-plan"[Title/Abstract] OR "F2"[Title/Abstract]) OR "Fertility diet"[Title/Abstract] OR "FODMAP"[Title/Abstract] OR "Hamptons"[Title/Abstract] OR "High Protein"[Title/Abstract] OR "HMR"[Title/Abstract] OR "Jenny Craig"[Title/Abstract] OR "keto*"[Title/Abstract] OR "LEARN"[Title/Abstract] OR ("low carb*"[Title/Abstract] OR "carbohydrate restr*"[Title/Abstract] OR "restricted carb*"[Title/Abstract]) OR ("low fat"[Title/Abstract] OR "fat free"[Title/Abstract] OR "fat restrict*"[Title/Abstract] OR ("restrict*"[All Fields] AND "lipid"[Title/Abstract])) OR "low glycemic index"[Title/Abstract] OR ("low salt"[Title/Abstract] OR "low sodium"[Title/Abstract] OR "salt restrict*"[Title/Abstract] OR "saltless"[Title/Abstract] OR "sodium free"[Title/Abstract]) OR "Mayo clinic diet"[Title/Abstract] OR "McDougal"[Title/Abstract] OR "Mediterranean"[Title/Abstract] OR "MIND"[Title/Abstract] OR "Nordic diet"[Title/Abstract] OR "Nutrisystem"[Title/Abstract] OR "Ornish"[Title/Abstract] OR "Okinawa"[Title/Abstract] OR "Optavia"[Title/Abstract] OR "paleo*"[Title/Abstract] OR "Pioppi"[Title/Abstract] OR "Plant-based"[Title/Abstract] OR "Portfolio diet"[Title/Abstract] OR "Pritikin"[Title/Abstract] OR "Protein power"[Title/Abstract] OR "Rosedale"[Title/Abstract] OR "Rosemary Conley"[Title/Abstract] OR "Salisbury"[Title/Abstract] OR "Scarsdale"[Title/Abstract] OR "slimming world"[Title/Abstract] OR "South beach"[Title/Abstract] OR "Stillmann"[Title/Abstract] OR "Sugar busters"[Title/Abstract] OR ("TLC"[Title/Abstract] OR "Therapeutic lifestyle changes"[Title/Abstract]) OR "Vegetarian"[Title/Abstract] OR "vegan"[Title/Abstract] OR "Volumetrics"[Title/Abstract] OR "Western diet"[Title/Abstract] OR ("weight watcher*"[Title/Abstract] OR "weightwatcher*"[Title/Abstract]) OR "ZONE"[Title/Abstract] OR "American Heart Association"[Title/Abstract] OR "Cardiac diet"[Title/Abstract] OR "Cardioprotective"[Title/Abstract] OR "Dietary guidelines"[Title/Abstract] OR "Dietary index"[Title/Abstract] OR "eating pattern*"[Title/Abstract] OR "eating plan*"[Title/Abstract] OR "Guidance"[Title/Abstract] OR "Healthy diet index"[Title/Abstract] OR "Healthy diet score"[Title/Abstract] OR "Nutrition therapy"[Title/Abstract] OR "Nutritional therapy"[Title/Abstract] OR "Prudent diet"[Title/Abstract] OR "Usual diet"[Title/Abstract]) AND ("diet"[MeSH Terms] OR "diet*"[Title/Abstract] OR "nutrition*"[Title/Abstract]) AND ("coronary disease"[Title/Abstract] OR "coronary heart disease"[Title/Abstract] OR "coronary artery disease"[Title/Abstract] OR "ischemic heart disease"[Title/Abstract] OR "myocardial infarction"[Title/Abstract] OR "angina"[Title/Abstract] OR "cardiac arrest"[Title/Abstract] OR "PCI"[Title/Abstract] OR "CABG"[Title/Abstract] OR "Percutaneous coronary intervention"[Title/Abstract] OR "coronary artery bypass graft"[Title/Abstract] OR ("cerebrovascular disease"[Title/Abstract] OR "stroke"[Title/Abstract] OR "cerebral haemorrhage"[Title/Abstract] OR "cerebrovascular accident"[Title/Abstract]) OR ("peripheral arterial disease"[Title/Abstract] OR "peripheral artery disease"[Title/Abstract]) OR ("AAA"[Title/Abstract] OR "abdominal aortic aneurysm"[Title/Abstract]) OR ("cardiovascular disease"[Title/Abstract] OR "CVD"[Title/Abstract] OR "vascular diseases"[MeSH Terms])) AND ("randomized controlled trial"[Publication Type] OR "controlled clinical trial"[Publication Type] OR "randomized controlled trial"[Title/Abstract] OR "Clinical trial"[Title/Abstract])

TABLE S1 Baseline characteristics of studies included in the systematic review and network meta-analysis

Study	Population	Country	Study follow-up (weeks)	Intervention diet		Reference diet		Sources of funding
				Description	Behavioral support	Description	Behavioral support	
**Ball, 1965**	264 males, <65 y, recently recovered from first MI	United Kingdom	159	Reduced fat. Low-fat dietary regimen with <40 g fat / day. Reduced caloric intake for overweight patients.	NR	Usual diet. Reduced caloric intake for overweight patients.	NR	Supported by the research committee of the North West Regional Hospital Board and by the Medical Research Council.
**Oslo Diet Heart study, 1970**	412 males, 30-64 y with first MI 1-2 prior inclusion.	Norway	260	Moderate carb. Cholesterol lowering diet low in animal fats and dietary cholesterol, rich in vegetable oil.	NR	Usual diet.	NR	NR
**Brown, 1984**	50 patients (26% female) with confirmed PAD of the lower extremities, symptoms of intermittent claudication, nonexistent or stable CHD and no insulin requirement.	Canada	52	Moderate carb. Low cholesterol, modified fat American Heart Association Hyperlipidemia Diet C. Individualized to achieve optimal weight. Limit alcohol as much as possible and restrict salt intake	4 days of intense training with a relative in small group sessions.	Reduced fat. High fiber, low cholesterol, very low fat diet based on the Pritikin maintenance diet. Individualized to achieve optimal weight. Limit alcohol as much as possible and restrict salt intake.	4 days of intense training with a relative in small group sessions.	Supported by the Medical Services Research Foundation of Alberta and the Special Services and Research Committee, University of Alberta Hospital, Edmonton, Alberta, Canada.
**Lyon Heart study, 1994**	605 patients (9.3% female), <70 y with MI < 6 months prior inclusion.	France	117	Mediterranean. Increased bread, root and green vegetables, fish. Less meat (beef, lamb, and pork to be replaced with poultry), no day without fruit. Butter and cream to be replaced with provided margarine. Moderate alcohol consumption in the form of wine was allowed.	1 h dietary counselling session by cardiologist and dietician. Reinforced after 8 weeks, then annually.	Usual diet. No dietary advice apart from that of hospital dieticians or attending physicians.	NR	Supported by grants from INSERM (Reseau Clinique), Ministry of Research (Aliments 2000 and 2002), CNAMTS, CETIOM, and ONIDOL, Astra-Calve BSN, and the Fondation pour la Recherche Medicale.
**Huh, 1996**	14 patients (16% female) with angiographically documented CAD.	South Korea	52	Reduced fat. Low-fat, low-cholesterol diet. Fat <10% of daily caloric intake, cholesterol <50 mg/d. Caloric restriction. Instructed to eliminate alcohol intake.	NR	Usual diet. Asked not to make dietary or lifestyle changes.	NR	

**Aquilani, 1999**	126 males with coronary heart disease and serum LDL-c above 3.37 mmol/L.	Italy	26	Reduced fat. Hypocaloric diet with energy intake equal to their resting energy expenditure. Wine consumption was optimal with 200 ml / d limit.	NR	Moderate carb. National Cholesterol Education Program (NCEP) Step 2. Hypocaloric diet with energy intake equal to their resting energy expenditure. Wine consumption was optimal with 200 ml / d limit.	NR	NR
**Sondergaard, 2003**	131 patients (30% female), 18-80 y with documented IHD (recent MI or unstable angina pectoris or stable angina pectoris and serum cholesterol ≥5 mmol/L.	Denmark	52	Mediterranean. Advice to eat ≥600 g of fruits and vegetables, modify fat intake from meat and dairy, eat fatty fish ≥1x per week, plenty of bread and cereals. Replace refined, hard animal margarine with vegetable oils.	Dietary advice by MSc in clinical nutrition. Advice based on 24-h recall.	Usual diet. Leaflet on healthy heart diet.	Single visit to dietician, not included in the study.	Supported by AstraZeneca
**Frost, 2004**	55 volunteers, aged between 30-70 years with a history of myocardial infarction, unstable angina or angiographically proved CAD.	United Kingdom	12	Healthy eating advice with weight loss advice if indicated and the use of at least one LGI food (<85 reference to white bread) at each meal.	One-to one counselling supported with regular visits and telephone calls.	Healthy dietary pattern based on the guidelines advocated by the COMA panel (1994). Target energy intake: 50% carbohydrate and 35% from fat.	One-to one counselling supported with regular visits and telephone calls.	NR
**Lindeberg, 2007**	29 males with WC > 94 cm and increased blood glucose. One of following conditions: acute coronary syndrome, history of MI, PCI or CABG or angiographically diagnosed coronary stenosis.	Sweden	12	Reduced fat. Paleolithic diet based on lean meat, fish, fruits, leafy and cruciferous vegetables, root vegetables (restricted amounts of potatoes), eggs and nuts. Advised not to consume >1 glass of wine. Avoid beer.	NR	Mediterranean. Consensus diet based on whole-grain cereals, low-fat dairy products, potatoes, legumes, vegetables, fruits, fatty fish and fats rich in monounsaturated fatty acids. Advised not to consume >1 glass of wine.	NR	Study was funded by Region Skåne and Lund University.

**THIS-DIET trial, 2008**	101 patients (26% female) <6 weeks after first MI.	United States of America	104	Mediterranean. Reduce saturated fat calories ≤7% and cholesterol to ≤200 mg/day and increase omega-3 fatty acids and mono-unsaturated fatty acids. Recommended increased intake of fruit, vegetables and whole grains with an emphasis on cold-water fish (3-5 times / wk) and oils from olives, canola and soybeans. Caloric restriction for patients with BMI > 25.	Individual counselling sessions. 6 different group sessions focused on behavioral modification and practical aspects of the assigned diets.	Reduced fat. Reduce saturated fat calories ≤7% and cholesterol to ≤200 mg/day. Recommended increased intake of fruit, vegetables and whole grains. Caloric restriction for patients with BMI > 25	6 different group sessions focused on behavioral modification and practical aspects of the assigned diets.	Supported by a Nutrition Grant from the Washington State Attorney General Vitamins Settlement Fund and intramural or in-kind support from the investigator's sponsoring institutions, The Heart Institute Spokane and Providence Medical Research Center, Sacred Heart Medical Center, Spokane, Washington, and Deaconess Medical Center, Spokane, Washington.
**Weber, 2012**	122 patients (34% female) with established or previous atherothromboti c CVD and at least one additional risk factor.	Brazil	182	Moderate carb. Brazilian cardioprotective diet. Avoid high energy dense food. Culturally accepted diet. Caloric restriction for patients with BMI > 25. 2000 mg/d of sodium was recommended	Weekly training sessions with dieticians.	Mediterranean. Usual diet with Mediterranean components. Caloric restriction for patients with BMI > 25. 2000 mg/d of sodium was recommended	Weekly training sessions	Funded by the Brazilian Ministry of Health (Programma Hospitais de Excelencia a Serviço do SUS).
**AUSMED Heart study, 2018**	56 adult patients (16% female) with CHD (acute MI, angina pectoris with documented CAD, CABG or PCI.	Australia	52	Mediterranean. Daily intake of extra virgin olive oil, nuts, vegetables, fruit and wholegrain cereals, regular intakes of legumes, fish and yogurt and limited intake of commercial sweets or pastries and red or processed meat. Moderation of poultry, eggs and feta cheese. For participants choosing to consume alcohol, red wine was suggested to drink in moderation (1-2 glasses /d) with meals.	3 face-to-face counselling sessions and 5 telephone counselling sessions.	Reduced fat. Daily intake of grains and cereals (mostly whole grains, 5-7 servings /day), vegetables (5-6 servings /day), fruit (2 servings / day), high-protein foods (2-3 servings /day) and low-fat dairy (2 servings /day.	3 face-to-face counselling sessions and 5 telephone counselling sessions.	This work was supported by La Trobe University (Understanding Disease RFA Start-Up Grant, 2013, the Australian Government Research Training Program Scholarship and a Northern Health PhD Scholarship and the United States National Institute for Diabetes, Digestive and Kidney Diseases (grant no. R44DK103377).
**BALANCE, 2019**	2534 Patients, aged 45 years or older with established CVD (CAD, CeVD or PAD)	Brazil	182 (median follow-up)	Macronutrient intake: 50%-60% of energy from carbohydrates, 10%-15% from proteins, 25%-35% from total fat. A cookbook of regional Brazilian modified recipes (o reduce SFA, dietary cholesterol, and sodium concentration) was also devised and given to the	Individual sessions with a registered dietitian every 6 months for 2 years.	Dietary pattern in accordance with current guidelines for CVD	Folder with foods that should be preferred or avoided	Funded by Hospital do Coração (HCor) as part of the "Hospitais de Excelência a Serviço do SUS (PROADI-SUS)" Program, in partnership with the Brazilian Ministry of Health

				participants as an educational tool.				
**DISCO-CT study, 2019**	91 patients (38% female) with stable coronary artery disease	Poland	26	DASH is focused on reduced sodium intake.	Six dietary counselling sessions within 6 months	Standard of care in accordance with the 2013 ESC Guidelines on the Management of Stable Coronary Artery Disease. No specific dietary counseling.	NR	This work was supported by a grant (2.15/III/15) from the Institute of Cardiology in Warsaw, Poland.
**CORDIOPREV, 2020**	805 patients (8% female) aged 20-75 with established CHD, without clinical events in <6 months	Spain	52	Mediterranean. Mediterranean diet, abundant use of virgin olive oil, vegetables, fresh fruit, legumes, fish, fresh nuts and seeds, reduction in meat, avoid additional fats.	Intensive dietary counselling. Personal interviews at baseline and at 6 months. Group education sessions 4x per year.	Reduced fat. Low fat diet, recommended by the National Cholesterol Education program and the AHA.	Intensive dietary counselling. Personal interviews at baseline and at 6 months. Group education sessions 4x per year.	The CORDIOPREV study was supported by the Fundacion Patrimonio Comunal Olivarero, Consejerı´a de Economı´a, Innovacio´n, Ciencia y Empleo and Ministerio de Ciencia e Innovacio´n, integrated into the framework ofthe National Plan For Scientific Research,Technological evelopment and Innovation 2013-2016, co-financed by the Instituto de Salud Carlos III (ISCIII) of Spain and also by the Directorate General for Assessment and Promotion of Research and the EU's European Regional Development Fund (FEDER). It was also partly supported by the U.S. Department of Agriculture (
**Von Haehling, 2013**	524 patients (26% female) with manifest CAD and MBS	Germany	26	Moderate carb. Tibetan diet, high-protein, and vitamin-rich food, preferably cooked and warm food.	Personal dietary and behavioral advice according to assigned diet.	Moderate carb. AHA prudent Western diet, balanced carbohydrate, low-fat, dietary fiber food, fresh fruit and vegetables (steamed or raw), polyunsaturated fatty acids.	Personal dietary and behavioral advice according to assigned diet. one calls	The study was supported by the German Cardiac Society (DGK) molecular imaging of atherosclerotic plaques' and in part by Klinische Forschergruppe KFO274 – 'Platelets, Molecular Mechanisms and Translational Medicine'
**Singh, 1992**	406 patients (10% female) with acute MI, possible acute MI or unstable AP.	India	52	Moderate carb. AHA healthy heart diet + additional fruits, vegetables, pulses, nuts. Mainly vegetarian diet with eggs 4-5 times / week and meat 1-2 times / wk.	Dietary advice was regularly enforced	Moderate carb. AHA healthy heart diet. Mainly vegetarian diet with eggs 4-5 times / week and meat 1-2 times / wk	Left to usual care after initial advice.	NR

AHA = American Heart Association, AP = Angina pectoris, BMI = Body mass index, CABG = Coronary artery bypass graft, CAD = Coronary artery disease, carb = Carbohydrate, CETIOM = Centre Technique Interprofessionnel des oléagineux Métropolitains, CHD = Coronary heart disease, CNAMTS = French National Health Insurance Fund for Salaried Workers, COMA = Committee on Medical Aspects of Food and Nutrition Policy, CVD = Cardiovascular disease, DASH = Dietary approaches to stop hypertension, ESC = European Society of Cardiology, IHD = Ischemic heart disease, INSERM = Institut national de la santé et de la recherche médicale, LDL = Low-density lipoprotein, LGI = Low glycemic index, MBS = Metabolic syndrome, MI = myocardial infarction, MSc = Master of science, NR = Not reported, ONIDOL = Organisation Nationale Interprofessionnelle des Oléagineux , PAD = Peripheral arterial disease, PCI = Percutaneous Coronary Intervention, SFA = Saturated fatty acid

FIGURE S1 Risk of Bias assessment

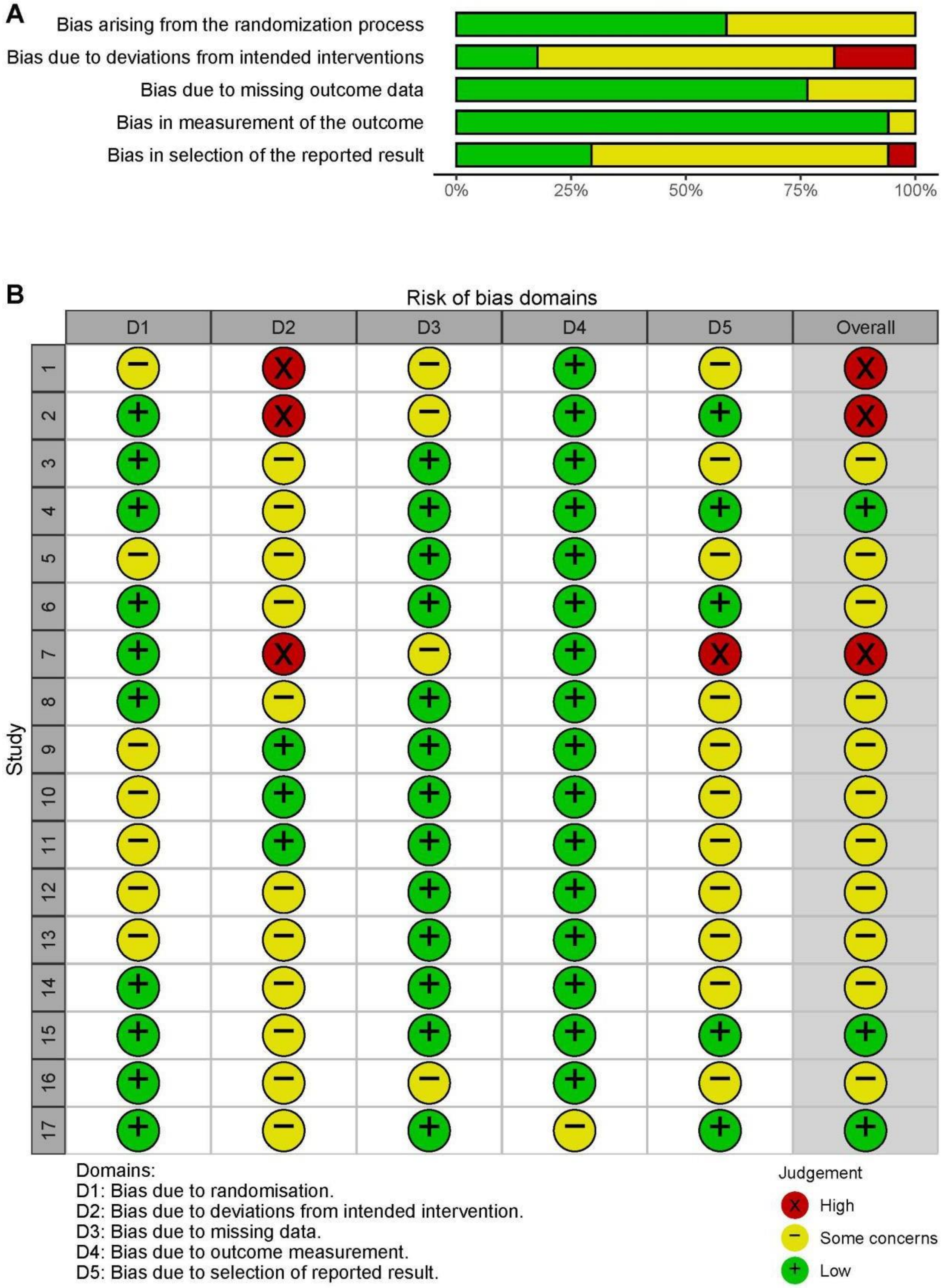

Risk of bias was determined using the Cochrane risk of Bias 2 tool. Study numbers refer to: 1: Aquilani 1999, 2: AUSMED Heart study, 3: Ball 1965, 4. BALANCE, 5: Brown 1984, 6: CORDIOPREV trial, 7 DISCO-CT trial, 8: Frost, 2004, 9: Huh 1997, 10: Lindeberg 2007, 11: Lyon Heart study, 12: Oslo diet heart trial, 13: Sondergaard 2003, 14: THIS-DIET trial, 15: Weber 2012, 16: Singh 2013, 17: Von Haehling 2013.

FIGURE S2 Network plots for short-term outcomes

For each endpoint, the number of clinical trials assessing the endpoint and number of participants are presented. The node sizes represent the number of participants randomized to a dietary pattern, and edge thickness is proportionate to the number of trials with a direct comparison between two dietary patterns.
RCT: randomized controlled trial, GI: glycemic index, carb: carbohydrate, HDL: high-density lipoprotein

Figure S2a Body mass index

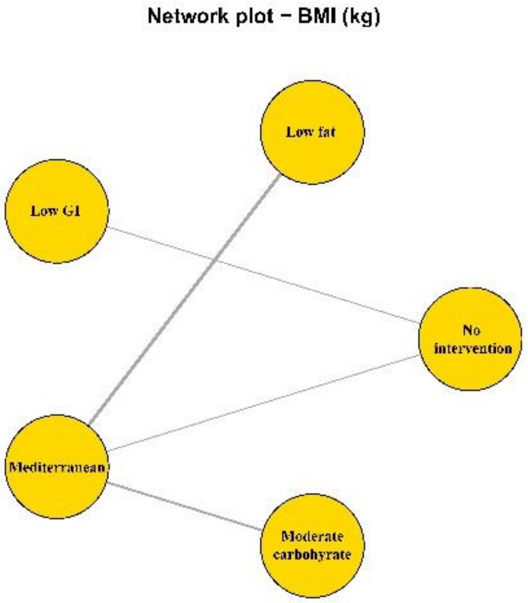

Figure S2b Total choltesterol

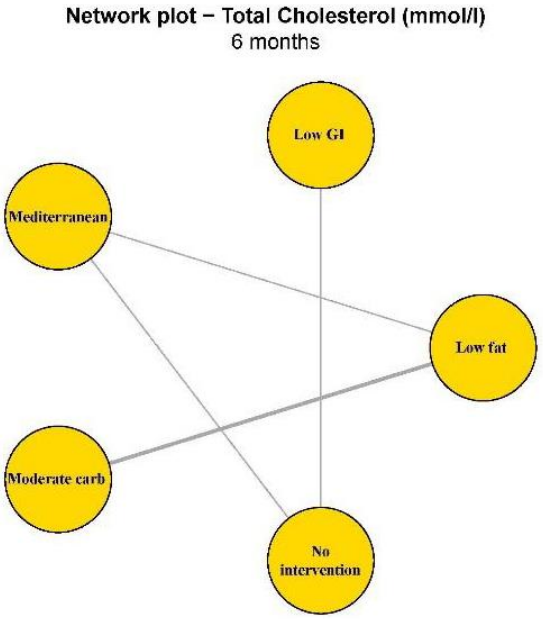

Figure S2c HDL-cholesterol

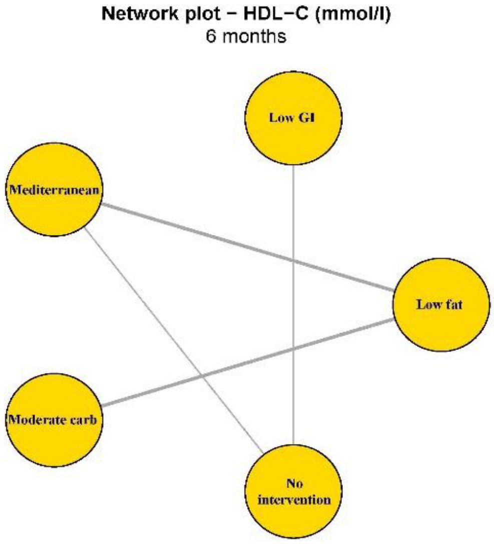

Figure S2d Triglycerides

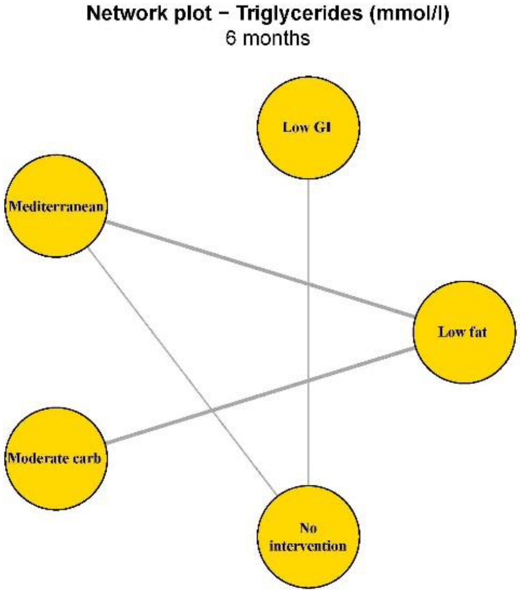

Figure S2e C reactive protein

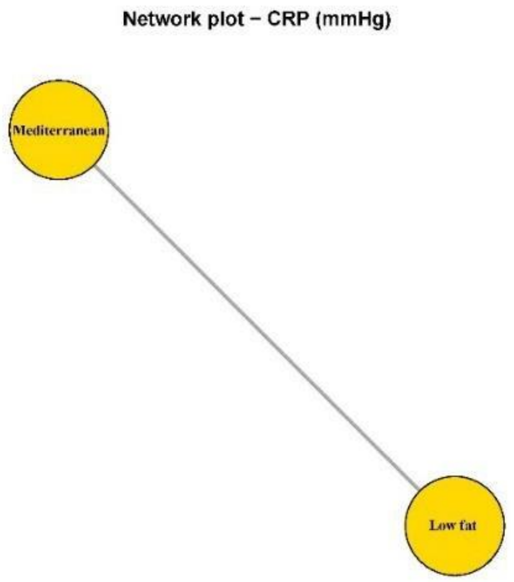

FIGURE S3 Network plots for long-term outcomes

For each endpoint, the number of clinical trials assessing the endpoint and number of participants are presented. The node sizes represent the number of participants randomized to a dietary pattern, and edge thickness is proportionate to the number of trials with a direct comparison between two dietary patterns.
RCT: randomized controlled trial, GI: glycemic index, carb: carbohydrate, HDL: high-density lipoprotein

Figure S3a Body weight

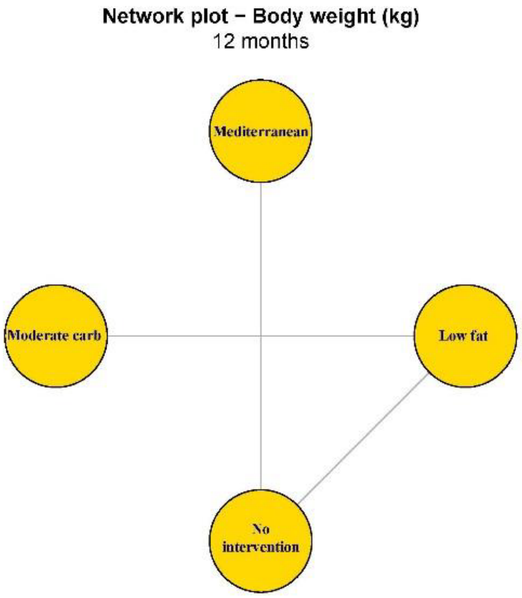

Figure S3b Body mass index

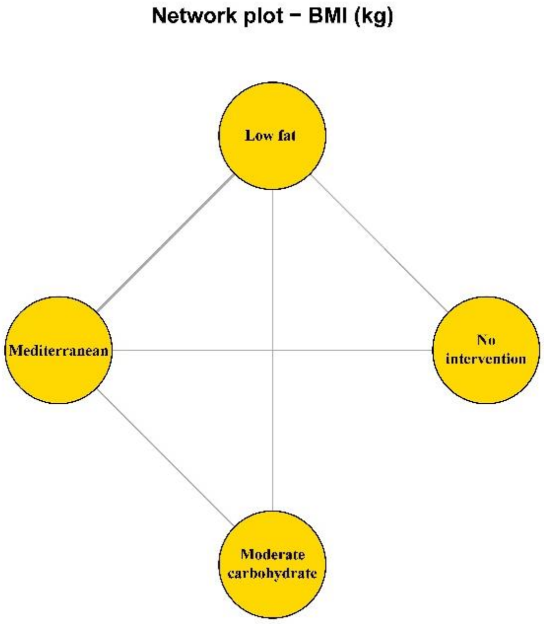

Figure S3c Systolic blood pressure

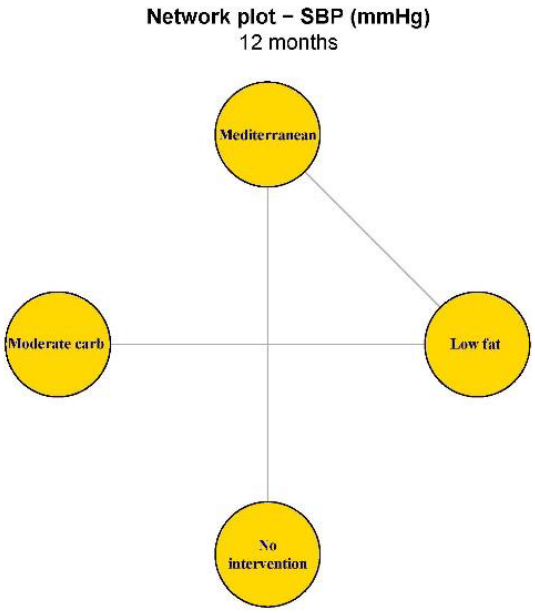

Figure S3d Total Cholesterol

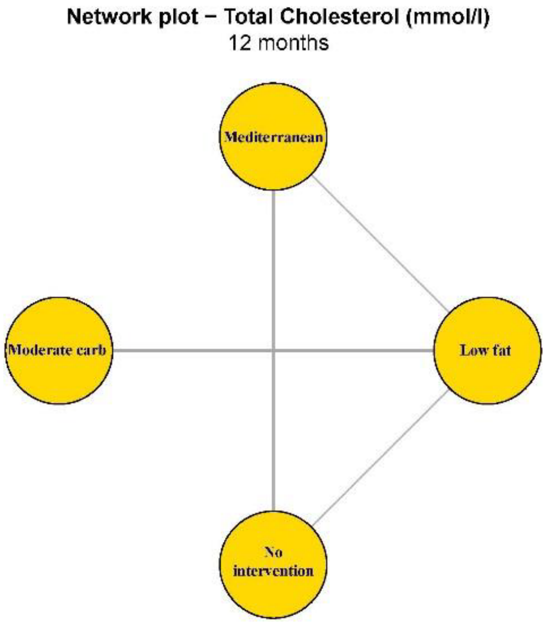

Figure S3e LDL-Cholesterol

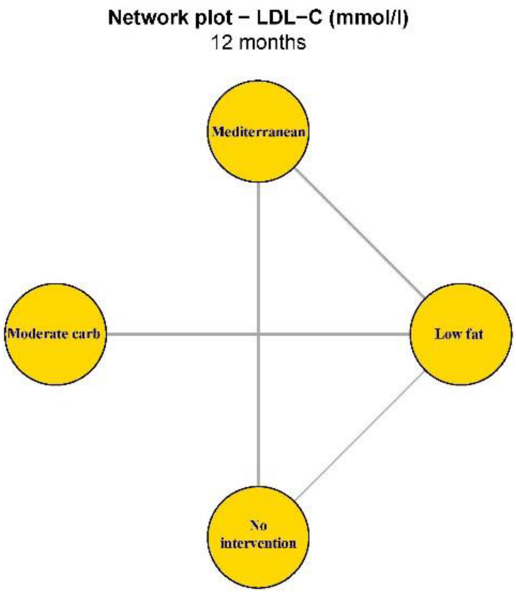

Figure S3g HDL-cholesterol

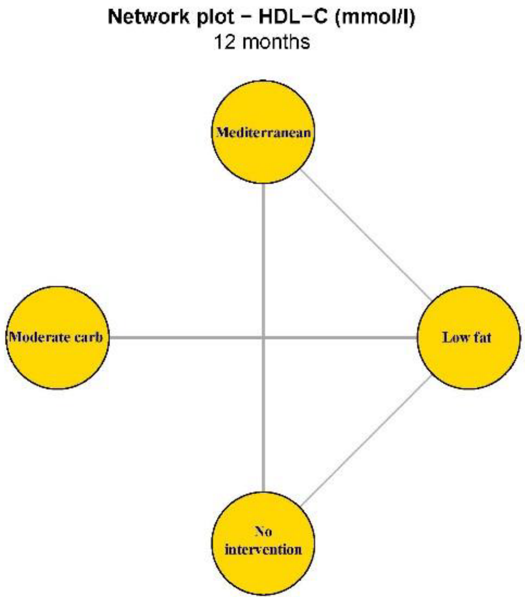

Figure S3g Triglycerides

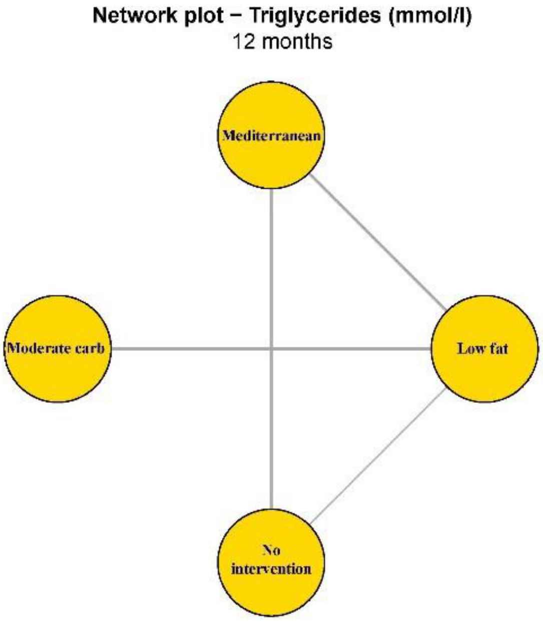

Figure S3h C reactive protein

FIGURE S4 League tables of the network estimates for the short- and long- term effects of dietary pattern on cardiovascular risk factors

Values correspond to the mean difference in reduction and corresponding 95% credibility interval in the outcomes for the dietary pattern in the column compared to the dietary pattern in the row. Values below and left of the dietary patterns represent changes at 6 months after diet initiation and values above and right of the dietary patterns represent changes at least 12 months after diet initiation.

Carb: carbohydrate, GI: glycemic index, LDL: low-density lipoprotein, 95%CrI: 95% Credibility interval

Figure S4a Mean differences in body weight (kg)

Mean difference (95%CrI) after 12 months

Mean difference adter 6 months (95% CrI)

**Low fat**	*NA*	3.6 (-8.9, 16.1)	-1.9 (-8.8, 5.0)	4.2 (-7.0, 15.4)
1.9 (-34.1, 37.3)	**Low GI**	NA	NA	NA
2.2 (-13.5, 17.7)	0.3 (-33.6, 34.3)	**Mediterranean**	-5.5 (-19.7, 8.6)	0.6 (-5.0, 6.1)
7.2 (-8.4, 22.8)	5.4 (-28.2, 39.4)	5.0 (-11.7, 21.7)	**Moderate carb**	6.1 (-7.1, 19.3)
2.6 (-20.6, 25.1)	0.6 (-26.2, 28.1)	0.4 (-19.8, 21.0)	-4.6 (-25.1, 15.8)	**No intervention**

Figure S4b Mean differences in body mass index (kg/m^2)

Mean difference (95%CrI) after 12 months

Mean difference adter 6 months (95% CrI)

**Low fat**	*NA*	-0.1 (-0.8; 0.5)	-0.1 (-0.9; 0.7)	0.4 (-0.6; 1.4)
-0.5 (-2.9; 2.0)	**Low GI**	*NA*	*NA*	*NA*
0.0 (-1.0; 0.9)	0.4 (-1.9; 2.7)	**Mediterranean**	0 (-1.0; 1.0)	0.5 (-0.3; 1.3)
0.4 (-2.0; 2.9)	0.8 (-2.4; 4.2)	0.4 (-1.8; 2.7)	**Moderate carb**	0.5 (-0.7; 1.7)
-0.2 (-1.6; 1.3)	0.3 (-1.8; 2.4)	-0.1 (-1.2; 1.0)	-0.5 (-3.1; 2.0)	**No intervention**

Figure S4c Mean differences in systolic blood pressure (mmHg)

Rows: **Mean difference adter 6 months (95% CrI)**; Columns: **Mean difference (95%CrI) after 12 months**

**Low fat**	*NA*	-3.0 (-9.2, 3.3)	1.4 (-2.6, 5.3)	-3.0 (-10.6, 4.7)
2.3 (-12.6, 18.2)	**Low GI**	*NA*	*NA*	*NA*
1.2 (-2.5, 6.8)	-0.9 (-15.6, 13.9)	**Mediterranea n**	4.4 (-3.0, 11.6)	0.0 (-4.5, 4.4)
7.4 (-0.3, 16.3)	5.1 (-11.2, 21.6)	6.0 (-0.9, 13.0)	**Moderate carb**	-4.4 (-12.9, 4.2)
0.2 (-7.2, 9.4)	-1.8 (-15.1, 11.3)	-1.0 (-8.0, 5.9)	-7.0 (-16.8, 2.7)	**No intervention**

Figure S4d Mean differences in total cholesterol (mmol/l)

Rows: **Mean difference adter 6 months (95% CrI)**; Columns: **Mean difference (95%CrI) after 12 months**

**Low fat**	*NA*	0.2 (-0.6, 1.5)	0.0 (-0.9, 1.0)	0.3 (-0.4, 1.8)
-0.5 (-1.6, 0.7)	**Low GI**	*NA*	*NA*	*NA*
-0.4 (-1.1, 0.3)	0.0 (-0.8, 0.9)	**Mediterranean**	-0.1 (-1.8, 1.0)	0.0 (-0.6, 1.0)
-0.1 (-0.5, 0.3)	0.3 (-0.8, 1.5)	0.3 (-0.5, 1.1)	**Moderate carb**	0.2 (-0.8, 2.1)
-0.6 (-1.4, 0.3)	0.0 (-0.8, 0.6)	-0.1 (-0.7, 0.4)	-0.4 (-1.4, 0.5)	**No intervention**

Figure S4e Mean differences in low density lipoprotein-cholesterol (mmol/l)

Rows: **Mean difference adter 6 months (95% CrI)**; Columns: **Mean difference (95%CrI) after 12 months**

**Low fat**	NA	0.0 (-0.4, 0.7)	0.0 (-0.6, 0.6)	0.0 (-0.4, 1.0)
0.1 (-1.0, 1.1)	**Low GI**	NA	NA	NA
0.0 (-0.4, 0.3)	0.0 (-1.0, 0.8)	**Mediterranean**	0.0 (-0.9, 0.7)	0.0 (-0.3, 0.7)
-0.5 (-0.9, 0.0)	-0.6 (-1.6, 0.5)	-0.5 (-1.0, 0.1)	**Moderate carb**	0.0 (-0.6, 1.2)
0.0 (-0.7, 0.7)	0.0 (-0.8, 0.6)	0.0 (-0.6, 0.7)	0.6 (-0.4, 1.4)	**No intervention**

Figure S4f Mean differences in high density lipoprotein-cholesterol (mmol/l)

Mean difference (95%CrI) after 12 months

Mean difference adter 6 months (95% CrI)

**Low fat**	NA	0.0 (0.0, 0.1)	0.0 (0.0, 0.1)	0.1 (0.0, 0.1)
0.0 (-0.3, 0.2)	**Low GI**	NA	NA	NA
0.0 (-0.1, 0.1)	0.0 (-0.2, 0.2)	**Mediterranean**	0.0 (-0.1, 0.1)	0.0 (0.0, 0.1)
0.1 (-0.0, 0.2)	0.1 (-0.2, 0.4)	0.1 (0.0, 0.2)	**Moderate carb**	0.0 (-0.1, 0.1)
0.0 (-0.2, 0.1)	0.0 (-0.2, 0.1)	0.0 (-0.2, 0.1)	-0.1 (-0.3, 0.1)	**No intervention**

Figure S4g Mean differences in triglycerides (mmol/l)

Mean difference (95%CrI) after 12 months

Mean difference adter 6 months (95% CrI)

**Low fat**	*NA*	-0.1 (-0.7, 0.4)	0.0 (-0.5, 0.9)	-0.0 (-0.7, 0.7)
0.5 (-1.7, 2.7)	**Low GI**	*NA*	*NA*	*NA*
0.2 (-0.3, 0.7)	-0.3 (-2.5, 1.9)	**Mediterranean**	0.2 (-0.6, 1.2)	0.1 (-0.4, 0.7)
-0.2 (-0.7, 0.3)	-0.7 (-3.0, 1.6)	-0.4 (-1.1, 0.3)	**Moderate carb**	-0.1 (-1.1, 0.8)
0.2 (-1.8, 2.3)	-0.3 (-1.2, 0.6)	0.0 (-2.0, 2.0)	0.5 (-1.7, 2.6)	**No intervention**

Figure S4h Mean differences in triglycerides (mmol/l)

Mean difference (95%CrI) after 12 months

Mean difference adter 6 months (95% CrI)

**Low fat**	*NA*	*NA*	*NA*	*NA*
NA	**Low GI**	*NA*	*NA*	*NA*
0.3 (-0.6, 1.4)	*NA*	**Mediterranean**	*NA*	*NA*
NA	*NA*	*NA*	**Moderate carb**	*NA*
NA	*NA*	*NA*	*NA*	**No intervention**

FIGURE S5 Short term effects SUCRA values for all outcomes

Figures represent the ranking of the different dietary patterns for the corresponding outcome. A higher ranking means that a dietary pattern is more likely to affect the outcome favourably, which was defined as reduction for all outcomes but HDL-cholesterol (where increase is the favourable outcome). Surface under the cumulative ranking curve (SUCRA) summarizes the probability of being the best treatment option into one number, with a value ranging between 0 and 1. A higher SUCRA values indicates that a treatment option performs better than alternatives, but effect size is not taken into account.
SUCRA: Surface under the cumulative ranking curve, 95%CrI: 95% credibility interval

Figure S5a Body weight – 6 months

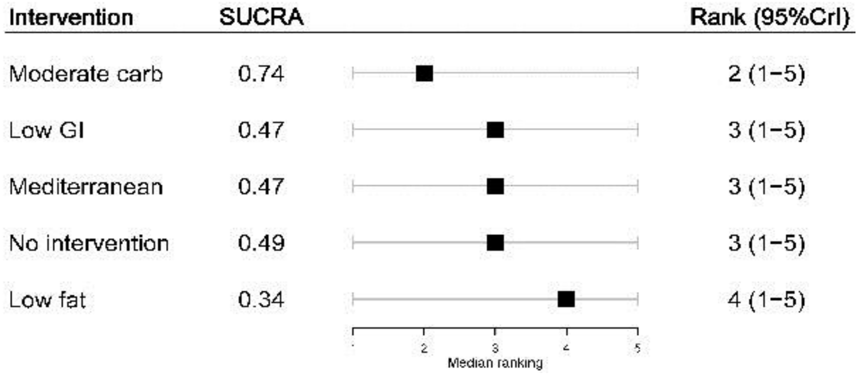

Figure S5b Body weight – 12 months

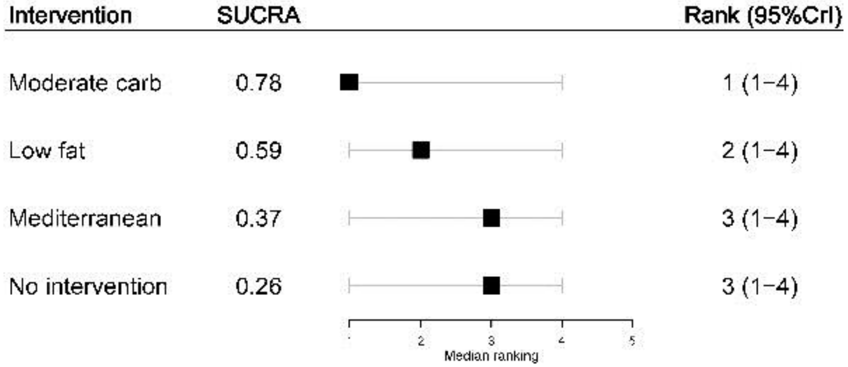

Figure S5c Body mass index - 6 months

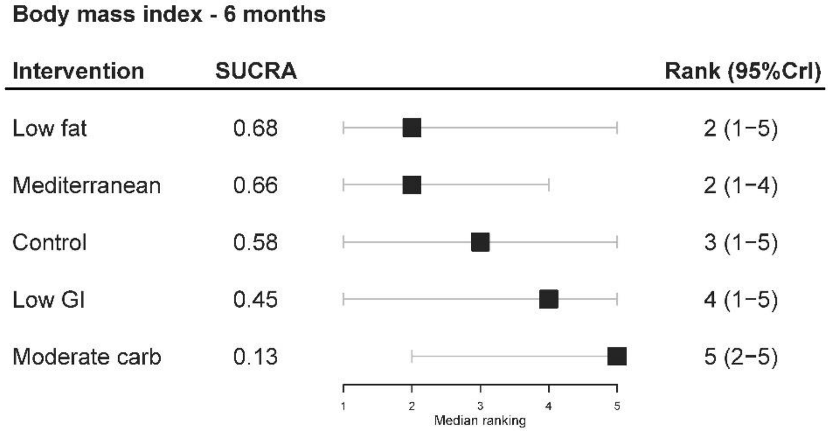

Figure S5d Body mass index – 12 months

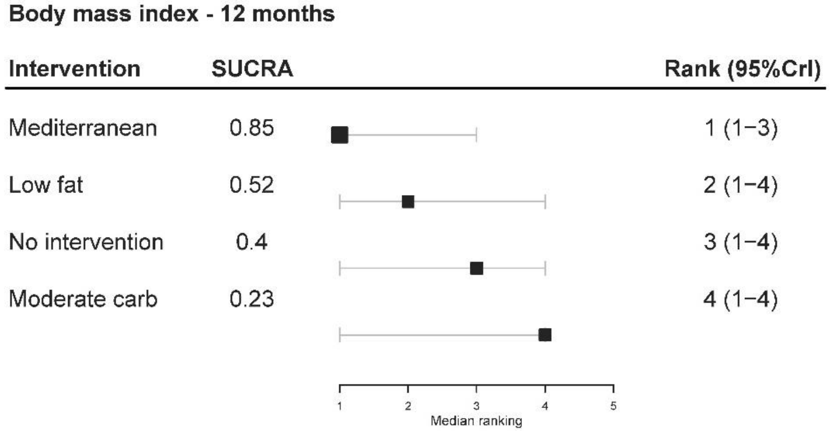

Figure S5e Systolic blood pressure – 6 months

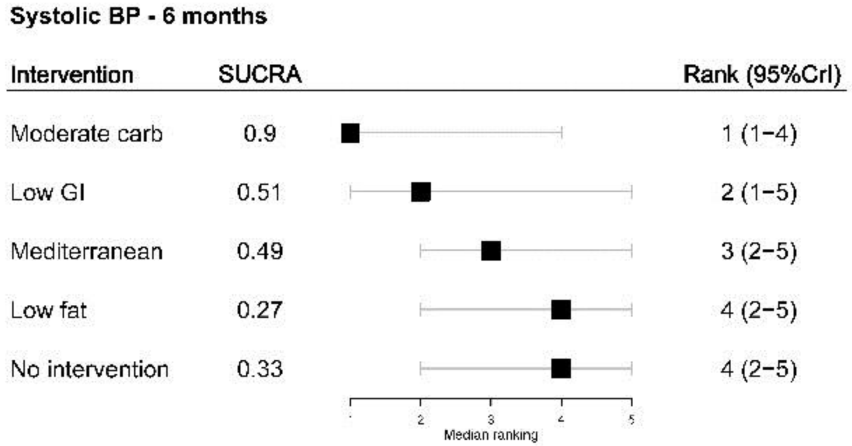

Figure S5f Systolic blood pressure- 12 months

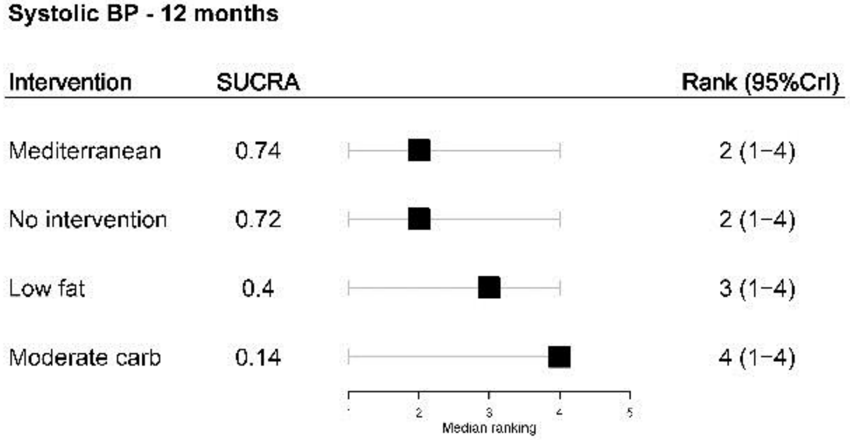

Figure S5g Total cholesterol = 6 months

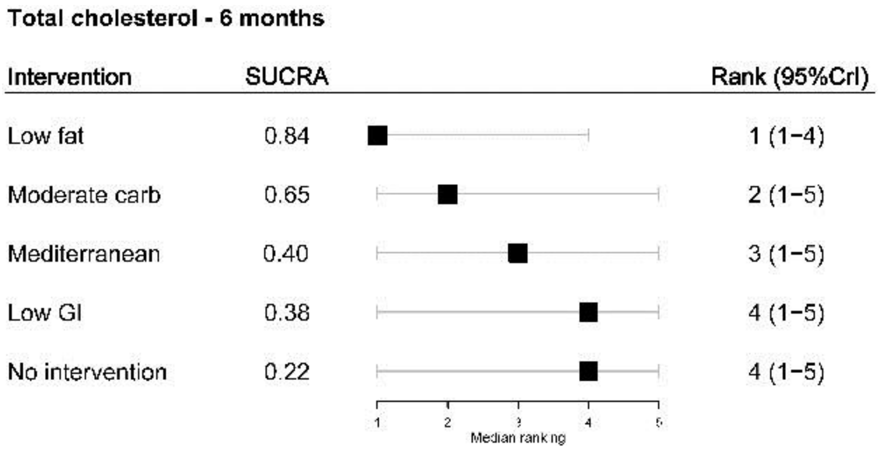

Figure S5h Total cholesterol – 12 months

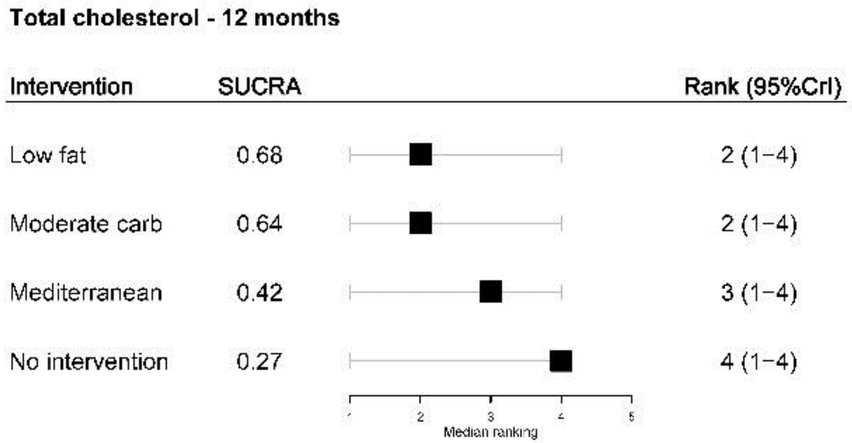

Figure S5i LDL-cholesterol – 6 months

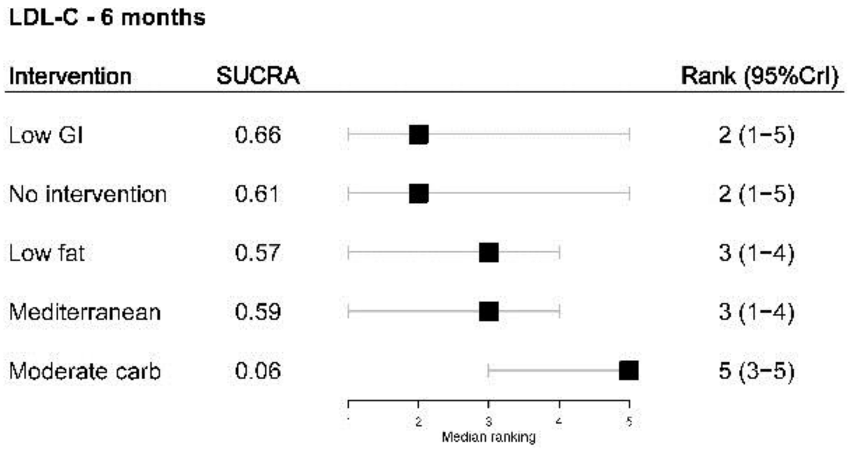

Figure S5j LDL-cholesterol - 12 months

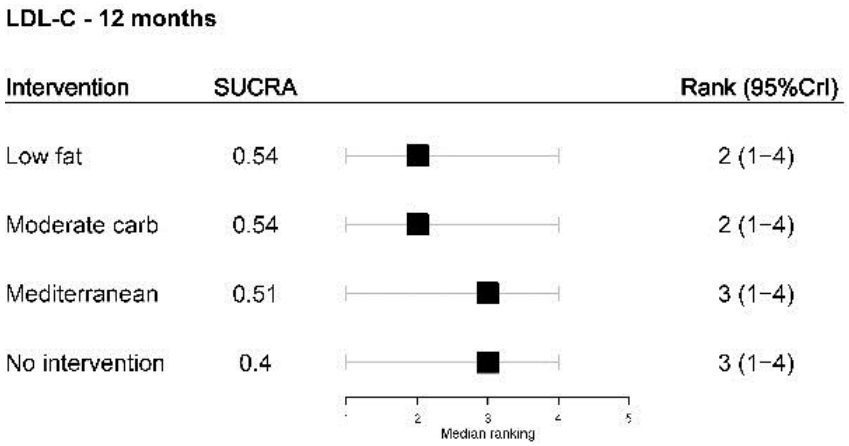

Figure S5k HDL-cholesterol = 6 months

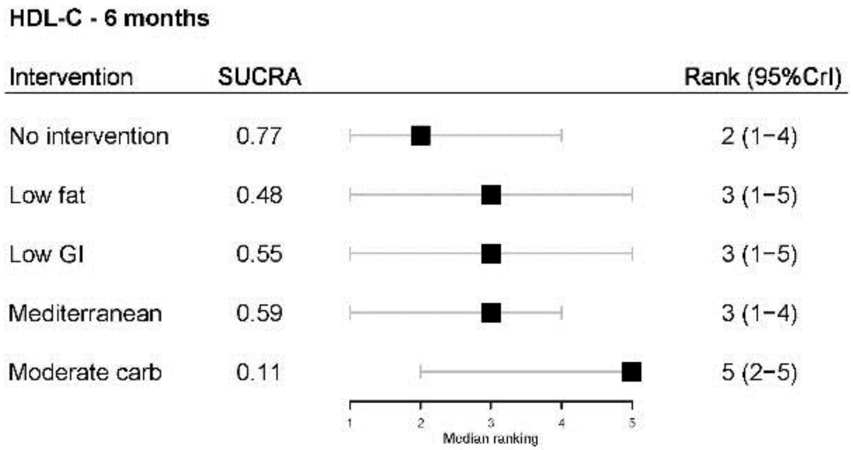

Figure S5l HDL-cholesterol – 12 months

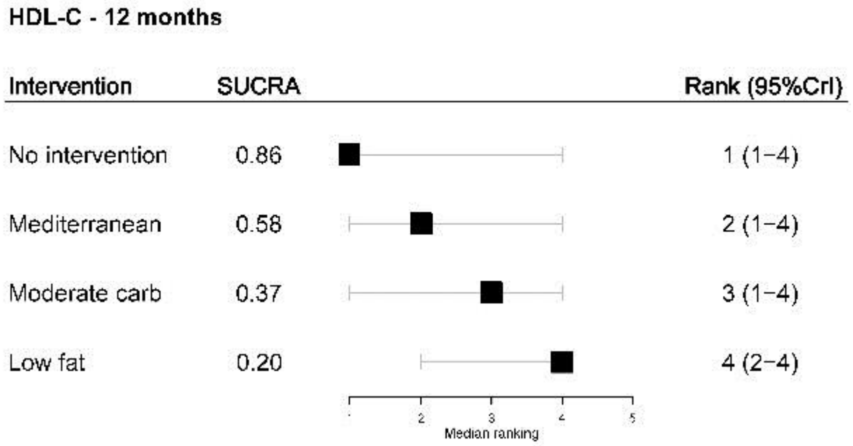

Figure S5m Triglycerides – 6 months

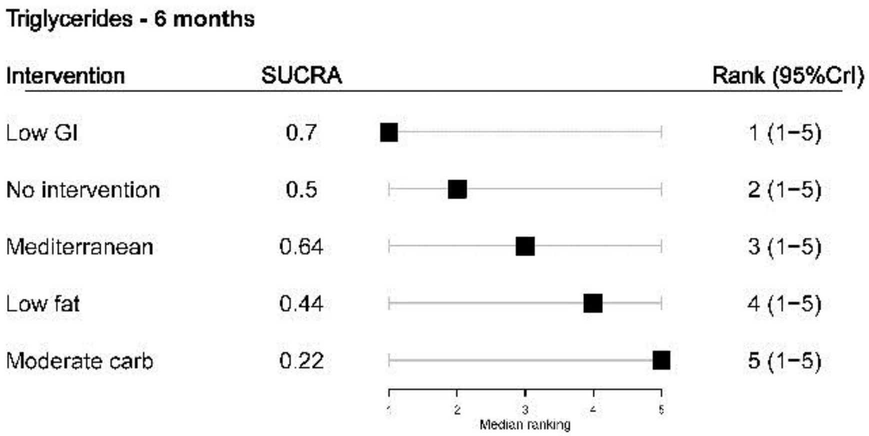

Figure S5n Triglycerides - 12 months

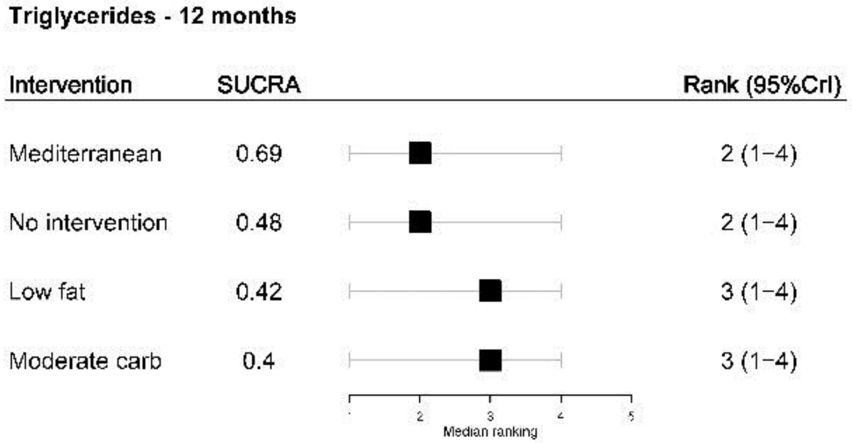

Figure S5o C reactive protein - 6 months

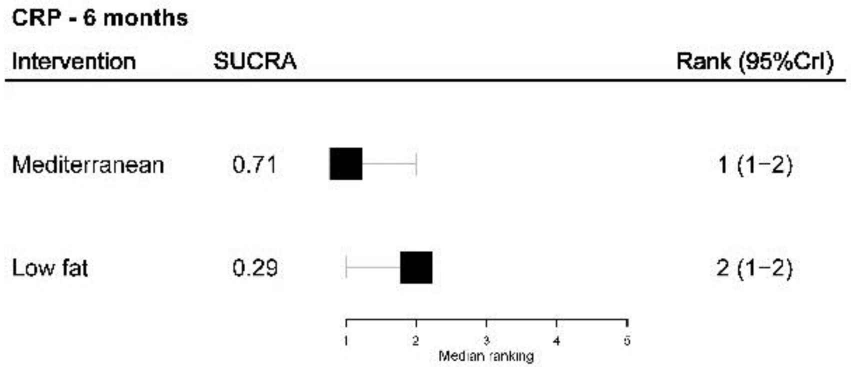

Figure S5p C reactive protein – 12 months

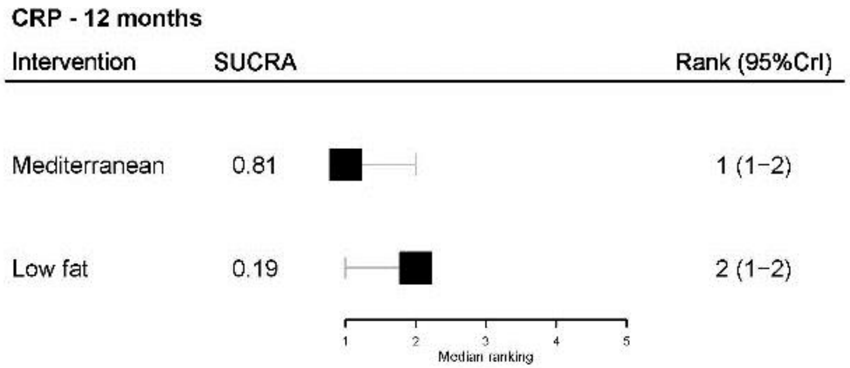

FIGURE S6 Sensitivity analysis: 6-month effects on body weight, systolic blood pressure and LDL-C in studies published in or after 2000

Intervention diet	Reference	RCTs	N	MD (95%CrI)
**Body weight (kg)**				
Low fat	No intervention			−2.7 (−9.8; 4.4)
Low GI	No intervention	1	55	0.8 (−7.0; 8.4)
Mediterranean	No intervention			−2.5 (−8.8; 3.9)
Moderate carb	No intervention	1	81	−3.7 (−8.1; 0.8)
**Systolic BP (mmHg)**				
Low fat	Mediterranean	3	206	1.2 (−2.5; 6.8)
Moderate carb	Mediterranean	1	122	6.0 (−13.0; 0.9)
**LDL-C (mmol/l)**				
Low fat	Mediterranean	3	206	0.0 (−0.4; 0.3)

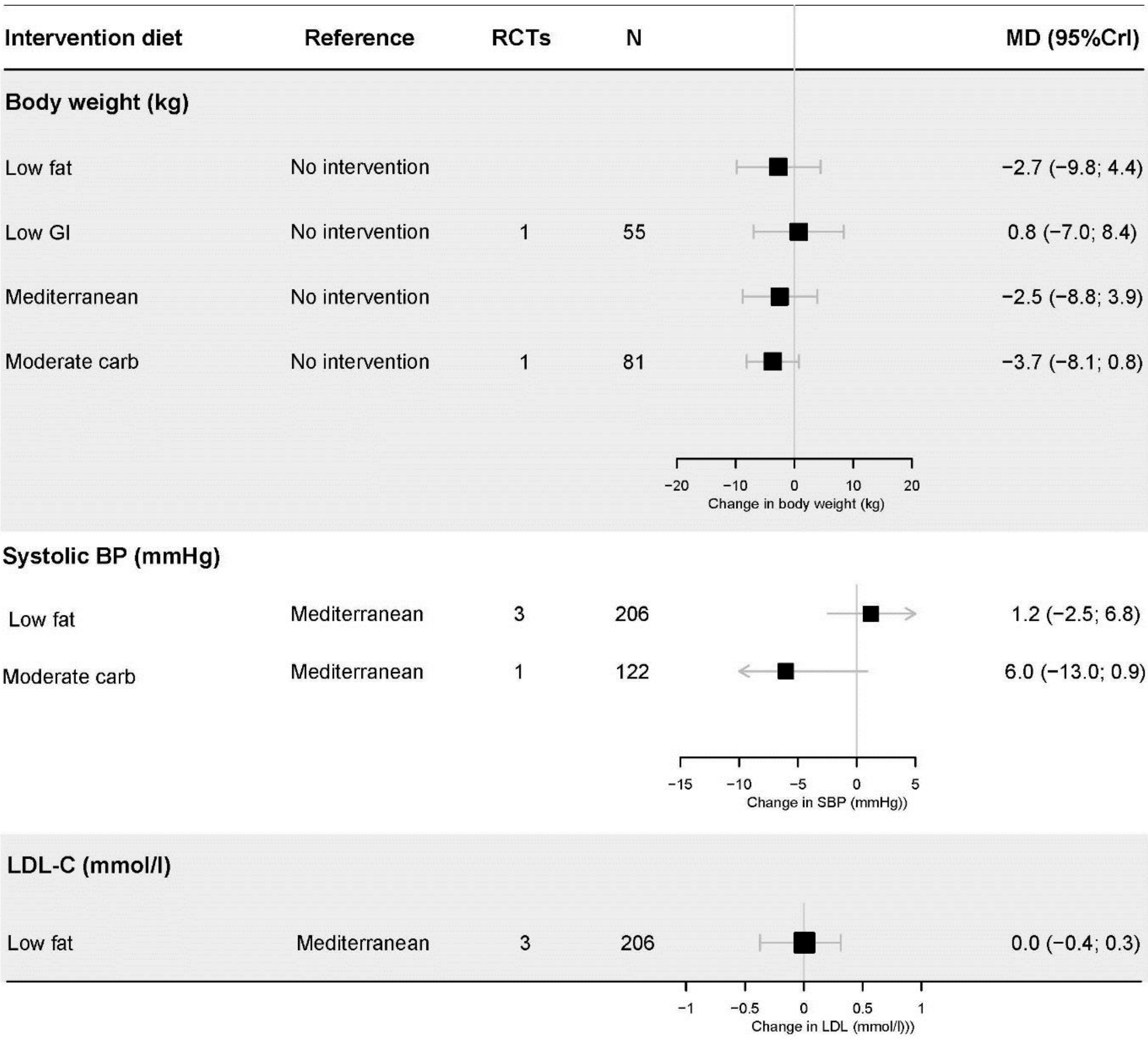

This table presents network estimates for the relative effects on primary outcomes based on studies published from the year 2000 onwards.. The column 'Direct comparisons' present the number of time a comparison was trialled and the 'N' column presents the total number of participants included in these clinical trials. Zero direct comparisons means that the presented network estimate is based on indirect evidence only.

Abbreviations: SBP: systolic blood pressure, LDL-C: low-density lipoprotein cholesterol

FIGURE S7 Sensitivity analysis: Comparison of short- and long term effects on primary outcomes

Intervention	Reference	Direct Comparisons	N
**Body weight**			
Mediterranean	Usual diet	1	558
Low fat	Usual diet	1	128
Mediterranean	Low fat	0	
**Systolic BP**			
Mediterranean	Usual diet	1	558
Low fat	Usual diet	0	
Mediterranean	Low fat	1	95
**LDL cholesterol**			
Moderate carb	Usual diet		
Mediterranean	Usual diet	1	558
Low fat	Usual diet		
Moderate carb	Low fat	1	46
Mediterranean	Low fat	1	95
Moderate carb	Mediterranean		

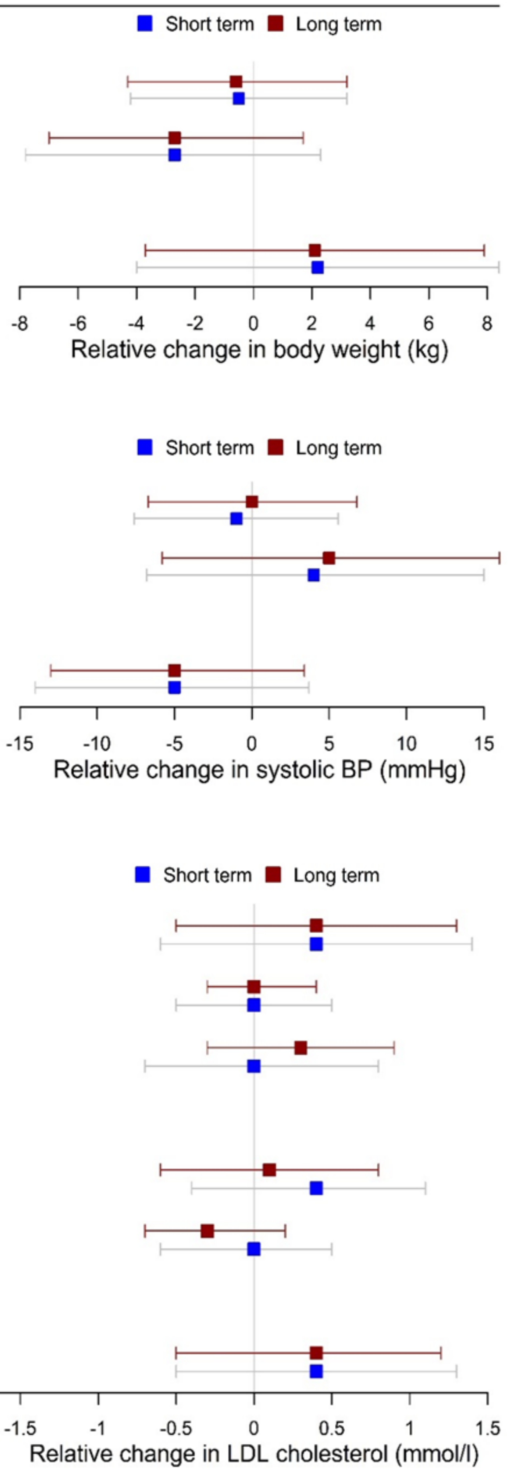

This table presents network estimates for the relative effects on primary outcomes based on studies that reported both short- and long-term effects. The short- and long-term effects are presented alongside each other. The column 'Direct comparisons' present the number of time a comparison was trialled and the 'N' column presents the total number of participants included in these clinical trials. Zero direct comparisons means that the presented network estimate is based on indirect evidence only. BP: blood pressure, LDL: low-density lipoprotein, moderate carb: moderate carbohydrate.

FIGURE S8 Sensitivity analysis - League tables for 6-month change in body weight, systolic blood pressure and LDL-cholesterol limited to CAD patients

FIGURE S8a Body weight (kg)

Mean difference adter 6 months (95% CrI)

**Low fat**				
1.8 (-1.5; 5.8)	**Low GI**			
-0.7 (-8.0; 6.6)	-2.6 (-10.8; 5.5)	**Mediterranean**		
1.2 (-1.6; 4.5)	-0.6 (-3.3; 1.9)	2.0 (-5.9; 10.0)	**Moderate carb**	
2.8 (-0.3; 5.8)	0.9 (-2.3; 3.6)	3.5 (-4.4; 11.4)	1.5 (-1.4; 4.1)	**No intervention**

FIGURE S8b Systolic blood pressure (mmHg)

Mean difference adter 6 months (95% CrI)

**Low fat**				
0.8 (-42.5; 43.7)	**Low GI**			
1.8 (-57.6; 60.2)	0.8 (-71.7; 75.7)	**Mediterranean**		
1.4 (-41.0; 43.8)	0.8 (-9.1; 10.9)	-0.1 (-74.3; 72.2)	**Moderate carb**	
8.2 (-54.8; 73.4)	7.7 (-40.4; 57.8)	6.6 (-80.3; 93.7)	6.8 (-40.0; 56.7)	**No intervention**

FIGURE S8c LDL-Cholesterol (mmol/l)

Mean difference adter 6 months (95% CrI)

**Low fat**				
-0.1 (-3.8; 3.6)	**Low GI**			
0.1 (-1.9; 2.1)	0.2 (-4.0; 4.3)	**Mediterranean**		
0.0 (-3.6; 3.6)	0.1 (-0.6; 0.8)	-0.1 (-4.1; 4.0)	**Moderate carb**	
-0.7 (-4.5; 3.2)	-0.6 (-1.7; 0.6)	-0.7 (-5.0; 3.6)	-0.7 (-2.0; 0.7)	**No intervention**

Values correspond to the mean difference in reduction and corresponding 95% credibility interval in the outcomes for the dietary pattern in the column compared to the dietary pattern in the row. Carb: carbohydrate, GI: glycemic index, LDL: low-density lipoprotein, 95%CrI: 95% Credibility interval

FIGURE 9 Sensitivity analysis - League tables for 6-month change in body weight, systolic blood pressure and LDL-cholesterol after exclusion of studies judged to be at high risk of bias

FIGURE S9a Body weight (kg)

Mean difference adter 6 months (95% CrI)

**Low fat**				
0.6 (-4.2; 5.5)	**Low GI**			
-0.6 (-7.4; 6.1)	-1.2 (-9.6; 7.0)	**Mediterranean**		
0.5 (-1.6; 2.7)	-0.1 (-4.5; 4.3)	1.1 (-5.9; 8.2)	**Moderate carb**	
1.7 (-1.6; 5.0)	1.1 (-4.0; 6.2)	1.1 (-5.9; 8.2)	1.2 (-1.3; 3.7)	**No intervention**

FIGURE S9b Systolic blood pressure (mmHg)

Mean difference adter 6 months (95% CrI)

**Low fat**				
-4.0 (-54.7; 45.8)	**Low GI**			
1.8 (-57.5; 62.1)	6.0 (-72.6; 83.8)	**Mediterranean**		
0.4 (-42.7; 42.3)	4.2 (-23.2; 31.5)	-1.7 (-73.9; 72.4)	**Moderate carb**	
7.6 (-55.9; 71.4)	11.9 (-43.8; 66.9)	5.7 (-80.4; 93.2)	7.4 (-40.9; 55.5)	**No intervention**

FIGURE S9c LDL-Cholesterol (mmol/l)

Mean difference adter 6 months (95% CrI)

**Low fat**				
0.3 (-3.5; 4.0)	**Low GI**			
0.1 (-1.8; 2.0)	-0.2 (-4.4; 4.0)	**Mediterranean**		
0.0 (-3.4; 3.4)	-0.3 (-1.7; 1.2)	-0.1 (-4.0; 3.9)	**Moderate carb**	
-0.1 (-4.9; 4.6)	-0.3 (-3.2; 2.5)	-0.2 (-5.3; 4.9)	-0.1 (-3.3; 3.0)	**No intervention**

Values correspond to the mean difference in reduction and corresponding 95% credibility interval in the outcomes for the dietary pattern in the column compared to the dietary pattern in the row. Carb: carbohydrate, GI: glycemic index, LDL: low-density lipoprotein, 95%CrI: 95% Credibility interval
